# A literature review of “lawful” text and data mining.

**DOI:** 10.12688/openreseurope.18013.1

**Published:** 2024-07-24

**Authors:** Giorgos Vrakas

**Affiliations:** 1 Law, University of Cyprus, Nicosia, Nicosia, Cyprus

**Keywords:** Text and data mining, copyright, data protection, contract law, human rights

## Abstract

Text and data mining (TDM) is a process, typically automated, that looks for patterns in data that may otherwise remain unnoticed. In a world where data driven solutions play a progressively more important role, TDM has become a vital tool in sectors ranging from medicine, to commerce, gaining widespread attraction. Nevertheless, a variety of regulatory frameworks not always specifically attuned towards regulating TDM continue to apply concurrently. The literature within the context of regulatory frameworks governing TDM is a fragmented piecemeal of valuable insights into what “lawful” TDM resembles.

This literature review adopts a grounded theory approach analysing 82 pieces of literature, collating views regarding “lawful” TDM, ultimately providing a holistic assessment of academics’ and practitioners’ views and opinions regarding the regulatory framework which governs TDM. A total of 7 categories were identified and each of these are analysed. Tables are provided in the Appendix (Refer to the extended data) highlighting which scholarly works were used for each section of the literature review, but also how those works were used.

It is ultimately concluded that the regulatory frameworks that apply to users conducting TDM are multifaceted, and ever-changing on a case-by-case basis. There is an ever-growing need for a holistic interpretation of the regulatory frameworks which apply, creating a map which would allow for users conducting TDM to navigate this complex web of legal rules.

## Plain language abstract

Text and data mining (TDM) is an automated method used to find hidden patterns in information and is often used to generate new knowledge. TDM is used widely today but the laws that apply to TDM are unclear. Similarly, research examining the laws that apply to TDM offer snippets of valuable insights.

This paper examines 82 pieces of literature which study the laws that apply to TDM, providing a more complete image of academics’ views on this topic.

What this paper shows is that the laws for people doing TDM are complicated and change depending on the situation. There is a growing need for a complete understanding of these laws, to help people doing TDM find their way through this tricky set of laws.

## Introduction

“ *Each day on Earth we generate 500 million tweets, 294 billion emails, 4 million gigabytes of Facebook data, 65 billion WhatsApp messages and 720,000 hours of new content added daily on YouTube*”. ^1^

This phenomenon has resulted in vast amounts of data being generated and stored and is accurately termed “big data”. In accordance with a specialist market and consumer analytics company, the total amount of data created, captured, copied, and consumed globally was 64.2 zettabytes in 2020, and is expected to grow to more than 180 zettabytes by 2025. ^2^

As per the European Commission “ *digital innovation, driven by the combination of Big Data, Cloud Computing, Mobile technologies and Social media, is one the most powerful drivers of change and the best opportunity for Europe to move back to a growth path*”. ^3^ Hence, the utilisation of big data is regarded as valuable to the European economy. Specifically, the capability to analyse “big data” is widely regarded as crucial in the context of decision making. ^4^ As per Gatti, data “ *is the new oil and lifeblood of economic development*” and just as oil needs to be burnt for it to be useful, raw data must be processed to realise its true potential. ^5^

Text and data mining (TDM) is a process, typically automated, that looks for patterns in data that may otherwise remain unnoticed, often transforming raw data into more understandable and useful information. ^6^ Generally TDM comes in a variety of different shapes and sizes varying on the type of data being used, data gathering techniques, analysis techniques employed, and presentation of data, but involves a common underlying objective. That is, utilising vast amounts of data to extract knowledge that would otherwise remain unnoticed. ^7^ Typically, TDM involves a series of stages such as document retrieval, document transformation (transforming the data into machine readable format), data extraction, (extracting the information which seems most useful), and finally mining that information resulting in new knowledge discovery. ^8^

In a world where data driven solutions play a progressively more important role, TDM has become a vital tool in sectors ranging from medicine, ^9^ to commerce, ^10^ gaining widespread attraction. Nevertheless, a variety of regulatory frameworks not always specifically attuned towards regulating TDM continue to apply concurrently. Just as the regulatory framework, the literature within the context of regulatory frameworks governing TDM is a fragmented piecemeal of valuable insights into what “lawful” TDM resembles. This literature review collates views regarding the regulatory frameworks governing TDM, providing a holistic assessment of academics’ and practitioners’ views and opinions regarding the regulatory framework which governs TDM.

This literature review forms part of a wider research project which aims to facilitate “lawful" TDM. This research will ultimately result in the creation of a practical guide and tool enabling users who conduct TDM to navigate the complex web of legal rules which make up the regulatory framework governing TDM.

To collate the relevant literature, keyword searches, using search operators were used on Google Scholar, Westlaw and LexisNexis. Namely, in October 2023 the keyword search used was ("Text and data mining") OR ((“Text” OR “Data”) AND ("mining" OR "mine")) AND (“law” OR “regulation” OR "legal"). This was updated periodically, up until May 2024 using the “sort by date” function identifying any newly published literature. Although lawful TDM is a relatively new topic of concern, this yielded a plethora of results. This is attributed to the returning of false positives such as mining in the geological sense. A total of 82 pieces of literature were collected and analysed manually adopting a grounded theory approach individually analysing and categorising them. For a list of all pieces of scholarly work analysed in this review please see Table 9 in the Appendix (Refer to the extended data).

As indicated by Table 1 below, a total of 7 distinct categories were identified. Literature was not always exclusively under one of the categories, meaning that some could fall under two or more of the categories. Tables for each of the category, listing which pieces of scholarly work were used is included in the Appendix (Tables 2 – 8) (Refer to the extended data).

**Table 1.** Categories of literature identified and the amount of literature within each of the categories.

Category	Description	Amount of Literature included in each section
Taxonomy of TDM	Identifying what TDM is and what it entails.	8
TDM and Intellectual Property Rights	How TDM interacts with Intellectual property rights, namely copyright and database rights.	42
TDM and Data Protection	How TDM interacts with data protection regulations.	12
TDM and Fundamental Rights	How TDM interacts with fundamental human rights	4
TDM and Contract Law	Hurdles for TDM caused by contracts and licenses.	11
TDM and Competition Law	How TDM interacts with competition law	2
Generalist Approach	A generalist approach where authors provide a more holistic approach toward the regulatory framework applicable to TDM	8

The structure of this literature review will follow the 7 categories identified. It begins by providing scholars’ views regarding what TDM entails, and how their view of TDM ultimately shapes their respective analysis of the regulatory frameworks. Subsequently, the jurisdictional variance of literature is highlighted, before moving onto presenting academics’ and practitioners’ views of the regulatory framework governing TDM, namely TDM’s interactions with intellectual property rights, data protection and privacy rules, fundamental human rights, contract law, and competition law. Finally, literature that decided to present the applicable regulatory frameworks more generally, are presented.

## A taxonomy of TDM

As aforementioned, TDM is a process, typically automated, uncovering knowledge from data that may otherwise remain unnoticed, often transforming raw data into more understandable and useful information. ^11^ In the literature, there is an undeniable articulation of the benefits that TDM has to offer, whether that be within the context of libraries, ^12^ scientific research and innovation, ^13^ medicine, ^14^ and generally its ability to yield economic advantages. ^15^ Wyner *et al.* , even introduce the idea of using TDM to extract information from case law databases thereby enhancing the legal profession, especially in common law systems which rely on case law. ^16^ Margoni argues that “ *TDM, together with other types of data-driven analytical tools, deserves its own autonomous methodological classification as computational legal methods*. ^17^ Nevertheless, there does not seem to be a common understanding of TDM amongst scholars.

Some scholars make a distinction between text mining and data mining. ^18^ Whilst this distinction may be vital on the technical side of TDM, altering the analysis techniques used, it arguably does not necessarily play a pivotal role in the regulatory frameworks which may apply. Data mining is sometimes seen as a subset of text mining since text mining is the computational process of analysing unstructured messy data, whilst data mining is the computational process of analysing structured data. ^19^

Other scholars focus on a taxonomy and categorisation of TDM as a way of explaining what regulatory frameworks a user conducting TDM might encounter. For instance, Colonna emphasises the fact that data mining involves separate processes, and in different contexts, each of them with varying legal implications. ^20^ Similarly, Tóth argues that the process of TDM involves several stages each of which could potentially conflict with regulatory frameworks. ^21^ Carroll also methodically works through each type of copying that happens in the course of collecting, formatting, processing, and storing data for TDM purposes, analysing relevant case law for each stage. ^22^ Fiil-Flynn *et al.* also claim that TDM projects potentially face regulatory restraints at different stages such as digitisation and compilation of data and the subsequent application of algorithms. ^23^ Equally, Rosati identifies three general TDM processes each of which with their own legal consideration:

“ *1) Access to content,*

*2) Extraction and/or copying of content, and;*

*3) Mining of text and/or data for knowledge discovery*”. ^24^

Likewise, Guadamuz and Cabell state that “ *it is difficult to generalise on what exactly the method for content mining is, as there are different algorithmic and model structures depending on the subject, the type of database, and the type of analysis being performed*”. ^25^ Nevertheless, the authors argue that most content mining roughly follows these steps:

“ *1. Individual content is created.*

*2. Content is placed into data set, repository or collection.*

*3. Miner gains access to the data.*

*4. Mining tools applied to the data set.*

*5. Analysis of the processed data.*

*6. New knowledge*”. ^26^

They then argue that legal conflicts will arise at stages 3 and 4. ^27^ Hence, it can be deduced that the literature although divided on the substantive procedural aspects of TDM stages, there is a cohesive undertone in the understanding that TDM involves a series of steps, each of which have their own legal considerations.

## Jurisdictional variance

Considering the nature of TDM, it is inevitable that users conducting TDM encounter multijurisdictional regulatory frameworks. As such, Flynn *et al.* call for a harmonised international approach towards the regulation of TDM, asking for the World Intellectual Property Organization (WIPO) to play a “ *constructive role in establishing a consistent international baseline*” for the regulation of TDM. ^28^ Nevertheless, as it stands users conducting TDM must navigate a complex web of regulatory frameworks spanning across multiple jurisdictions. Given the inherent difficulties of comprehensively examining the inner workings of lawful TDM on a global scale, this literature review will provide scholarly insights from the United States (US), European Union (EU), and United Kingdom (UK) providing insights where relevant from other jurisdictions. ^29^ Similarly, the practical guide and tool aimed at facilitating lawful TDM, created as part of this research’s wider purpose, will take into account and comparatively analyse these jurisdictions.

## Regulatory frameworks which govern TDM

Flynn *et al.* highlight the importance of being able to conduct TDM without disproportionate legislative barriers but argue that a legislative patchwork of regulatory frameworks spanning various jurisdictions may in fact hinder the progress of TDM. ^30^ This section aims to outline academics’ and practitioners’ views and opinions regarding the different regulatory frameworks which govern TDM.

A variety of regulatory frameworks are examined when discussing “lawful TDM” such as copyright and sui generis database rights, data protection and privacy, contract law, competition law and fundamental rights. Some scholars take a generalist approach examining a variety of frameworks which interact with the act of TDM whilst others adopt a more targeted approach examining one regulatory interaction. This review will present authors’ views systematically, presenting interactions with distinct regulatory frameworks separately. Subsequently, it will collectively discuss the views of authors who adopt a generalist approach.

### TDM and Intellectual Property Rights

Bearing in mind the definition of TDM presented above, it can be deduced that the act of TDM may conflict with intellectual property rights where the work or database being reused is protected by intellectual property rights. In the EU, such protection is afforded by Directive 2001/29 (InfoSoc Directive), ^31^ Directive 2009/24 (Software Directive), ^32^ or Directive 96/9 (Database Directive). ^33^ Other jurisdictions have their own regulatory frameworks for affording intellectual property rights. This section aims to present authors’ views, drawing insights from a variety of jurisdictions beginning with the EU approach, as this seems to make up the bulk of scholarly work. Namely 29 out of the 42 pieces of literature analysed under this category examined how TDM interacts with EU copyright and database rights.

It is often argued that since data itself is not protected by intellectual property rights, the act of TDM should in theory not interfere with any intellectual property rights. ^34^ Recital 9 of the Directive on Copyright in the Digital Single Market (CDSM) acknowledges this by stating that TDM “ *can also be carried out in relation to mere facts or data that are not protected by copyright, and in such instances no authorisation is required under copyright law. There can also be instances of text and data mining that do not involve acts of reproduction or where the reproductions made fall under the mandatory exception for temporary acts of reproduction provided for in Article 5(1) of Directive 2001/29/EC, which should continue to apply to text and data mining techniques that do not involve the making of copies beyond the scope of that exception”. ^35^* This however, is not always the case. In some instances, the act of copying involved with TDM will often encroach upon a rightsholders right to reproduce a work afforded to them through copyright protection. ^36^ For instance, when creative works are extracted, it is often the case that the entirety of that work is reproduced, resulting in infringement of the rightsholder’s exclusive right of reproduction. ^37^ Nasr *et al.*, were able to show that generative Artificial Intelligence (AI), which utilises data mining mechanisms sometimes regurgitates its training data in its entirety. ^38^ Similarly, Baris who examined derivative musical works created by AI, states that “ *where an AI-generated output reproduced protectable parts of a work that was used in the training of the AI system”,* this would likely amount to copyright infringement. ^39^ On the other hand Senftleben, argues that at an international copyright law level, the act of TDM is not prohibited. ^40^ Awarding copyright protection is ultimately a balancing exercise of all stakeholders’ interests. As will emerge from the discussion below, this balancing exercise becomes more complex in the realm of TDM.

The act of TDM may in some instances consist of an act of extraction and/or re-utilisation of a database protected in the EU by the sui generis database right. ^41^ As stated by Rosati “ *if the content extracted and/or copied is included in a database, then both copyright and the sui generis (database) right – these being two rights that are independent of each other and may subsist together at the same time on the same database) – might come into consideration”.* ^42^ Hence, depending upon the circumstances under which data is accessed, how data is processed and presented, it is possible that intellectual property rights are infringed.

The majority of scholarship in this domain emanates from the applicability of copyright exceptions to the act of TDM, most notably with the recent introduction of the two new mandatory copyright exceptions within the EU jurisdictions under Articles 3 and 4 of the CDSM. ^43^

In a pre-CDSM era, several Member States had already introduced their own TDM-specific copyright exceptions. The UK for instance, was the first Member State (in a pre-Brexit era) to have introduced their own TDM-specific exception back in 2014. ^44^ Section 29A of the Copyright, Designs and Patents Act 1988 introduced a text and data analysis exception for the purposes of non-commercial research. ^45^ Similarly, France introduced their own TDM exception applying to both works and databases. ^46^ Estonia and Germany also introduced their own TDM-specific exceptions. ^47^ Greece had also introduced its own rules regulating the act of web harvesting and archiving. ^48^ As per Geiger *et al.*, “ *this regulatory patchwork* […] *led to a fragmented legal environment in the EU*”, which ultimately led to the introduction of the CDSM exceptions so as to create a harmonised level playing field in the EU. ^49^ Jackson Bertón, who examined the applicable copyright frameworks of the five largest economies of South America in a pre-CDSM era, claimed that even then, the EU was falling behind other more competitive jurisdictions, but ultimately found that South American frameworks were not prepared for digital research techniques. ^50^

Scholars at this stage had called for the broadest possible EU TDM-specific exceptions so as to “ *boost the international competitiveness of its knowledge economy*”. ^51^ Once the dust had settled and the final version of the CDSM was published, Article 3 and 4 introduced two new mandatory exceptions arguably creating a new avenue for “lawful TDM”. Article 3 mandates Member States to allow for cultural heritage and research institutions to freely use protected works for scientific text and data mining research purposes. ^52^ Article 4 necessitates the introduction of a general TDM exception, allowing other entities to develop algorithms without the burden of authorisation and remuneration payment, provided the right has not been expressly reserved by the rightsholder. ^53^ These exceptions were welcomed to a certain extent, for their potential to harmonise a collective EU-wide approach towards “lawful TDM”, ultimately fostering competition. ^54^ For instance Papadopoulos *et al.*, who examined libraries’ views and opinions of web-harvesting (a form of TDM), and found that most libraries already conduct such forms of TDM and those that do not indicate a desire to do so, concluded that the EU CDSM TDM-specific exceptions create “ *a favourable legal foundation for the deployment of Web-harvesting and archiving operations through the national libraries of the EU Member States”*. ^55^

Nevertheless, authors have not strayed away from criticising these new set of mandatory EU TDM-specific exceptions. For instance, Ducato and Strowel present their arguments in a table which outlines all that these two exceptions got right, wrong, and also those sections which raise more questions. ^56^ They ultimately argue that Article 3 is too narrow with its limited scope of cultural heritage and research institutions whilst Article 4 is limited in the sense that it can be overridden by contracts. ^57^

This limiting of scope in Articles 3 and 4 of the CDSM to content that is “lawfully accessed” is interpreted in Recital 14 as including content that is openly accessible online and materials made available to users through the conclusion of a contractual agreement. ^58^ Whilst Article 3 cannot be overridden by contracts, Article 4 can. ^59^ This has resulted in commentators characterising this as giving rise to an opt-out mechanism. ^60^ The European Copyright Society, in its comment addressing selected aspects of the implementation of Articles 3 to 7 of the CDSM, argued that as a result of this provision “ *publishers might price TDM into their subscription fees and, as a consequence, few research organisations would be able to acquire licences for all databases that are relevant for a TDM research project*”. ^61^ It may even result in biased AI systems where it becomes more “ *economically attractive for developers to train their algorithms on older, less accurate, biased data, or to import AI models already trained on unverifiable data”.* ^62^ This, arguably greatly limits the applicability of the exception, putting the EU at a competitive disadvantage when compared to jurisdictions like the US which will be discussed in greater detail below, and undermines the “ *widespread assumption that the right to read should be the right to mine*”. ^63^ In a blog post, Keller argues that the way in which the proposed, at the time, EU AI Act is being phrased explicitly reinforces this opt-out mechanism, with some of the big names in generative AI also having shown evidence that they intend to implement some form of opt-out mechanisms for rightsholders. ^64^ Keller however, argues that this creates a fragmented framework for rightsholders who have to individually opt out of all new generative AI machines. ^65^ Nevertheless, the author paints the EU’s approach towards regulating AI, which inevitably regulates TDM as balanced, implying that this could in turn result in it being the next global standard. ^66^ Similarly, Senftleben argues that the EU AI Act could serve as a template for other regions promoting a global standard supporting human creativity in the age of AI. ^67^ Namely, they argue that the primary objective of the EU AI Act is to “ *ensure that authors are properly remunerated for the use of their works in AI training processes”. ^68^* They see this as a necessary step in order to support human creativity, thereby preventing the market from being overrun by AI-generated content. ^69^

Another issue that may be brought back to the limelight by this opt-out mechanism, is the interplay between copyright and technical protection measures (TPMs). TPMs allow for rightsholders to place architectural constraints on their works to regulate access. Under Article 6(1) of the InfoSoc Directive, TPMs are not to be circumvented, whilst under Article 6(4), rightsholders are to make exceptions for those uses which may be able to circumvent TPMs by falling under a copyright exception. ^70^ TPMs may be implemented as a way of utilising a rightsholder’s ability to opt out from TDM. ^71^ Nevertheless, this will undoubtably create uncertainty as to when TDM is being carried out for the purposes of research, which may inadvertently result in an over-blocking of TDM for research purposes. ^72^ A study conducted by the Max Planck Institute for Innovation and Competition suggests that whilst rightsholders should be able to utilise TPMs to restrict TDM, “ *such measures must not go beyond what is required, i.e. technical protection measures must be precluded from making TDM unnecessarily more difficult or even de facto impossible”*. ^73^ Havlikova who examined the interplay between the opt-out provision in Article 4 of the CDSM and web scraping, a form of data collection from web sources which is often the starting point of some forms of TDM, found that rightsholders may struggle to reserve their rights. ^74^ They attribute this to the fact that for content published online, a rightsholder’s reservation must be made using machine-readable means and “ *according to Recital 18 of the CDSM Directive, such machine-readable means may include metadata and terms and conditions of a website or a service*”. ^75^ They argue that as a result of such a wide and unstructured means afforded to a rightsholder allowing for them to reserve their right “ *could be the Achilles heel of the TDM exception”. ^76^* They ultimately argue that “ *Robots.txt, which is a simple text file containing rules on which crawlers may access which parts of a site”, ^77^* could be one feasible way of reserving one’s rights in a machine-readable means, but that this would also have its drawbacks, especially when it comes to indexing websites. ^78^ As it stands, the author calls for a more standardised method of reserving one’s rights through machine readable means. ^79^

As aforementioned, awarding copyright protection is a balancing exercise of all stakeholders’ interests. One way of balancing the extent of protection afforded is through the applicability of exceptions. These mandatory exceptions, whilst a step forward in the context of a harmonised approach towards the regulation of TDM, have their limitations which may ultimately tip the balance in favour of rightsholders. 

The limitation of scope of these TDM-specific EU exceptions has been regarded as a major pitfall in the EU’s attempt to become more competitive in the digital era. ^80^ For instance, Rosati argues that they “ *might have a negative impact on the (unlicensed) development of AI creativity”*. ^81^ As per Manteghi “ *the vague and narrow nature* [of the EU CDSM TDM-specific exceptions] *could force users to consider alternative solutions to avoid potential infringement claims”. ^82^* One of these solutions, Manteghi claims could be found under Article 35 of the proposed Data Act which “ *provides a safe harbour for users needing to access, use or share only databases made of data generated by the use of a product or related service (IoT data)”. ^83^* Whilst limited, to certain types of TDM which will utilise certain specific types of databases it is possible that users conducting TDM may find refuge under this proposed Article. Another alternative may be offered by Article 5(1) of the InfoSoc Directive which allows for transient copies to be made. ^84^ Nevertheless, as per Margoni and Kretschmer whilst Article 5(1) of the InfoSoc Directive “ *retains a significant potential for TDM activities and computational uses, the cumulative, occasionally narrow and partially uncertain nature of its conditions and the fact that it only covers temporary reproductions, does not offer a clear and comprehensive solution within which not only science but virtually any human activity employing text and data analytics can operate confidently”.* ^85^

The extent of these exceptions’ reach is inevitably dependent upon the national implementation of Member States. Pereira in their examination of the Portuguese implementation of these exceptions, which “ *has been faithful”* to the CDSM, argues that these exceptions seek to balance the interests of all relevant stakeholders. ^86^ Other commentators have however, called for a wider approach. For instance, Micke called for Finland to take an expansive transposition thereby guaranteeing the freedom to mine. ^87^ Similarly, Calabrese questions why the EU legislature did not exempt TDM users from infringing a rightsholder right of communication to the public in the TDM exceptions, and praises Italy for doing so in their national transposition. ^88^ Calabrese concludes by stating that the Italian approach “ *can be appreciated as an attempt of ‘better’—or at least ‘not worse’—harmonization in terms of free communication to the public for TDM, not forgetting that the EU has also to face the regulatory competition of third-country systems which are more open to innovation in this field*”. ^89^ When stating this they we referring to the Japanese approach which was the first nation in the world which introduced a TDM-specific copyright exception allowing for TDM researchers to utilise copyright protected works for machine learning purposes. ^90^ Dermawan similarly argued that EU Member States shall consider adopting the Japanese approach towards a more favourable TDM exemption in their national implementation of the CDSM. ^91^ Nevertheless, Rosati, when examining the extent to which national member states have freedom to diverge from Articles 3 and 4 of the CDSM, stated “ *both Articles 3 and 4 require a minimalistic transposition technique on the side of national legislatures, with the result that the normative content of either provision cannot be compromised at the Member State level. In turn, a national* [exception] *with a broader scope of application than what either provision allows would be incompatible with EU law”.* ^92^ At the time of writing this review, Poland had yet to implement the TDM-specific copyright exceptions into national law. The Polish government argues that their delayed implementation of the CDSM enabled a thorough examination of the implications of generative AI for copyright protection, ultimately deciding that training generative AI models on copyright protected works should fall outside the scope of the TDM-specific copyright exceptions. ^93^ How this rationale plays out remains to be seen, but as noted by Paul Keller in a blog post, it is “ *clear that any attempt to exclude from the scope of the TDM provision the reproductions made in the context of training generative AI models would, prima facie, result in a non-compliant implementation”*. ^94^ Hence, it is unlikely that such a divergence is accepted, but it does highlight the fact that national implementation of these exceptions remains fragmented. In their master’s thesis, André Stéphan, in analysing the national implementation of France, Germany, the Netherlands, Spain and Ireland argues that “ *if the objective was to create a digital single market, where stakeholders could benefit from the same rules in each Member State, it has not been reached”. ^95^* This implies that there is still a long way to go before true harmonisation is reached.

Another jurisdiction under which TDM researchers may find refuge is that of the US. There are numerous scholars which approach TDM through the lens of the US’s fair use copyright exceptions. Namely, as did EU scholars, several authors who examine the US’s regulatory framework for TDM assert that TDM is and should be lawful as a matter of US copyright law. ^96^ In other words such authors claim that the US fair use copyright exceptions would encompass the act of TDM. Most justify this reasoning by presenting the lawsuits that commercial authors and publishers lodged against Google, ^97^ and the HathiTrust Digital Library. ^98^ These cases involved the digitisation of printed books, allowing for users to search through this collection of literature. In accordance with the Author Guild’s perspective the systematic copying of copyright protected authorial works amounted to copyright infringement. However, Google and HathiTrust argued that their re-use was “fair use” as it was socially beneficial, but also since copyright protected works were not displayed in full. Only small extracts of the copyright protected works were presented to those who used the search function. These arguments ultimately swayed the court to rule in favour of Google and HathiTrust in finding that their reuse amounted to fair use.

Sag argues that TDM would have always found refuge under the US’s fair use exceptions and the US courts have now solidified this position. ^99^ Sag makes a distinction which the courts failed to do in these cases between a non-expressive use and transformative use. ^100^ Sag argues that TDM would fall under the category of a non-expressive use, whereby the act of TDM does not communicate original forms of expression for the purposes of them being read. ^101^ They assimilate it to photocopying a magazine and throwing it in a fireplace, which would not be regarded as infringing. ^102^ It is asserted that in Google Book saga cases non-expressive reuse was treated as a subset of transformative uses falling under “fair use”. ^103^

Carroll, argues that in the US, “ *a researcher can legally download all or a portion of the Sci-Hub collection* [a website which contains copious copyright infringing academic literature] *solely for TDM research*”. ^104^ Whilst, such an argument may attract greater controversy in that it tips the balance in favour of users to the detriment of rightsholders, it is actually supported by others. ^105^ In a recent blog post about an upcoming journal article, Thomas Margoni puts forward this same idea, but within the remits of an EU jurisdiction. ^106^ Margoni argues that the lawful access requirement found under Articles 3 and 4 of the CDSM which ultimately creates a TDM realm where content which is accessed needs a license (discussed in greater detail below), should “ *only cover the behaviour of the beneficiary of the exception and not extend to the status of the accessed source”. ^107^* Whether this liberal interpretation will indeed be the case remains to be seen.

Similar to its EU counterpart, the US allows for TPMs restricting TDM. ^108^ Nevertheless, Dombrowski and Tilton highlight the historical development of an exception for circumvention of TPMs for research purposes in US. ^109^ They state that “ *per the final decision, researchers residing in institutions of higher education could bypass DRM for research under a series of conditions. This included staff and students if they are a part of a research team or as a part of teaching. The university must own the source of the data (i.e., not accessing it via a subscription service), and the researchers must take “effective security measures” to protect the data. As a result, researchers at institutions of higher education can conduct TDM with sources such as DVDs and ebooks, which is a significant development for the Digital Humanities”*. ^110^ Nevertheless, they argue that while the ruling expands TDM access for “well-resourced” institutions, it falls short when considering wider TDM users and activities. ^111^

It can be deduced that authors generally view intellectual property rights as restricting one’s freedom to conduct TDM. Whilst the recent introduction of the EU CDSM TDM-specific copyright exceptions provides a new avenue towards “lawful TDM”, their narrow scope acts as a barrier to free mining. The wider approach of the US arguably creates a more welcoming market for TDM in the US. In a 2022 Research Paper Sean Flynn *et al.* categorised global copyright exceptions for research purposes focusing on TDM exceptions. ^112^ They provide an informative world map comprising of seven colours categorising countries as having a general research exception, restriction on sharing, restrictions to private reproductions, restrictions to institutional users, restrictions on types of works, TDM being restricted and countries that were not mapped. ^113^ They then comparatively analyse each of their categorisations. They also provide a figure comparing specific TDM exceptions that have been introduced. ^114^ They ultimately “show that although every copyright law in the world has at least one exception that promotes research purposes, there is a wide degree of variation between countries” ranging across the spectrum of categorisations they identified with some like the US, being more favourable than others. ^115^ As per Rosati “ *the US copyright regime has been considered more favourable to TDM practices than what appears to be the case under European laws”. ^116^* This is also evidenced by an empirical study conducted by Handke *et al.*, in 2015 who empirically examined bibliometric data from 43 large economies, including the 15 largest EU Member States, spanning from 1992-2014. ^117^ They found that data mining based articles were more common in countries where mining is “probably allowed”, under copyright law like that of the US, “ *which suggests that a more permissive copyright framework is associated with more data mining research”*. ^118^ On the other hand, Kretschmer *et al.*, who examined machine learning, natural language processing and computer vision for computer moderation as case studies where TDM is vital, found that legal uncertainties like those created by fair use exceptions, can lead to practices exploiting these uncertainties for commercial gain, particularly by large tech corporations. ^119^ Thus, the balancing of stakeholder interests in the realm where intellectual property rights and TDM intersect arguably still needs refining.

### TDM and Data protection

Given the inherent processing of data involved in the act of TDM, it is inevitable that users conducting TDM encounter data protection related regulatory frameworks. ^120^ Nonetheless, how TDM interacts with data protection frameworks seems under-researched. Colonna states that in existing literature “ *while the applicability of copyright law to TDM is being considered, there is very little research being done concerning the applicability of data protection law to this new technology”. ^121^* This section aims to nevertheless, provide an outline of existing research in the field examining the interplay between TDM and data protection regulatory frameworks.

One of the most influential regulatory frameworks in this regard is the EU General Data Protection Regulation (GDPR). ^122^ As per Dove “ *one might consider the GDPR to be a Europe-centric law of little global consequence […] on the contrary, the territorial scope of the GDPR follows the data that it protects and therefore has direct bearing on the activities of organisations based in countries around the world”.* ^123^

This cross-border reach of the GDPR may in fact restrict certain forms of TDM. As stated by Colonna, “ *the ability to conduct meaningful research utilising TDM requires not only being able to access data remotely but also being able to share and further process results within the Digital Single Market and beyond*” *. ^124^*

Whilst the GDPR, is generally viewed as an advancement in data protection law, encouraging responsible scientific research by introducing a plethora of stringent pre-processing and procedural requirements such as data protection by design, requirements of reporting data breaches, the appointment of data protection officers, the enforcing of hefty fines, and the right to be forgotten, it is argued that it does not necessarily represent a radical shift in data protection laws. ^125^ Nevertheless, it also argued that some businesses face challenges in going from GDPR non-compliance to compliance. ^126^ “ *Organisations and businesses have been processing personal data of people, in cases without explicit consent, up to the extent required for their businesses, retaining it for longer periods, and even subletting it to other businesses to be used for their own processing purposes*”. ^127^

In existing literature examining the interplay between TDM and data protection and privacy rules, a lot of emphasis is placed on privacy by design, or privacy preserving measures. ^128^ GDPR non-compliance may result in hefty fines being placed. ^129^ Hence, an emphasis on privacy preserving measures in existing literature may be correlated to an increasing need for GDPR compliance.

Privacy by design is enshrined under Article 25 of the GDPR which asks of data controllers and processors to “ *both at the time of the determination of the means for processing and at the time of the processing itself, implement appropriate technical and organisational measures, such as pseudonymisation, which are designed to implement data-protection principles, such as data minimisation, in an effective manner and to integrate the necessary safeguards into the processing in order to meet the requirements of this Regulation and protect the rights of data subjects*.” ^130^ It is often recommended that techniques for preserving privacy are adopted at early stages of a research project to align with respective regulatory frameworks. ^131^

A number of scholars in this domain, attempt to provide practical guides on how to implement privacy by design. For instance, Sangaroonsilp *et al.*, identified “ *an emerging need to translate complex privacy concerns set out in regulations and standards into requirements that are to be implemented in software applications”.* ^132^ They consequently “ *developed a comprehensive taxonomy of privacy requirements for software systems by extracting and refining requirements from the widely-adopted GDPR and ISO/IEC 29100 privacy framework as well as the newly developed Thailand PDPA and the region-specific APEC privacy framework*”. ^133^ Hence, they took a multijurisdictional approach to the applicability of data protection and privacy related regulatory frameworks developing a holistic taxonomy of the requirements a business which collects and/or processes data will have to adhere to. This could equally apply to a user conducting TDM. They broke these requirements down into 7 categories, including themes like lawfulness, purpose limitation, data minimisation, accuracy, storage limitation, integrity and confidentiality, and accountability, with a total of 71 requirements. ^134^

Other scholars provide more technical guidance on implementing a privacy by design approach. Clifton and Marks emphasise the need to balance between the benefits of data sharing and the risks associated with data mining, advocating for careful control of access to data to ensure efficient sharing without compromising security and privacy. ^135^ The authors acknowledge the evolving nature of the field and call for ongoing research to address emerging challenges in preventing unwanted data mining. ^136^

Schermer examines the data mining approaches in profiling which involves discovering correlations in databases to represent individuals or groups. ^137^ They explore two data mining approaches: descriptive, which uncovers relations between data objects without a specific target, and predictive, which predicts events based on established patterns. ^138^ Schermer identifies an number of privacy related risks associated with profiling using data mining techniques such as discrimination, de-individualization, and information asymmetries. ^139^ They argue that traditional privacy protection strategies alone are insufficient, and a multidisciplinary approach involving increased accountability, transparency, and algorithmic safeguards is crucial. ^140^ They argue that for descriptive data mining, awareness among data subjects and controllers about the possibilities, limits, and risks of data mining is essential. ^141^ Similarly predictive data mining requires transparency to prevent inaccurate applications, emphasizing the importance of distinguishing between direct and indirect indicators in decision-making. ^142^

Clifton *et al.*, highlight the growing interest in privacy-preserving data mining, but the existence of ambiguity in defining privacy. ^143^ They underscore the need for diverse measures of privacy preservation, providing four distinct measures for doing so. ^144^ First, the concept of bounded knowledge is introduced which involves data obscuration techniques where information about a protected attribute may be revealed within certain bounds. ^145^ They provide a metric for doing so which adds noise to certain variables. ^146^ Second the so-called “need-to-know” concept is introduced which implies that data should be processed only if necessary for a specific purpose, aligning with legal and privacy regulations. ^147^ Secure Multiparty Computation is suggested as a basis for data mining that adheres to the "need to know" standard. ^148^ “ *If the results of the data mining are required to accomplish an allowable task, then learning those results should be allowed under the “need to know” standard under which most privacy regulations allow release of information. Data mining approaches that are Secure Multiparty Computations can be proven not to disclose anything except the results”. ^149^* Third they argue that certain items, including individual data items and rules, may need protection from disclosure categorising such techniques for achieving this under the category of “protected from disclosure”. ^150^ Existing techniques in the database security community for inference prevention are discussed, but challenges in defining appropriate thresholds persist. ^151^ Finally they present anonymity techniques as a concept for privacy preserving data mining techniques. ^152^

Agrawal and Aggarwal aim to protect user information while still enabling data mining. ^153^ The proposed approach involves perturbing data values using a known distribution, allowing the reconstruction of aggregate distributions rather than individual records. ^154^ Similarly, in their book, Vaidya, Zhu and Clifton explore techniques such as data perturbation and Secure Multiparty Computation to address potential misuse of data. ^155^

Lindell and Pinkas address the challenge of two parties with private databases wanting to collaborate on computing a data mining algorithm without revealing the contents of their databases to each other. ^156^ The goal is for each party to only learn information from the output of the data mining algorithm. ^157^ They propose the use of decision tree learning. ^158^ One concern that is raised is the concept of the so called “semi-honest adversary”, where malicious parties follow the protocol but attempt to learn additional information from the communication transcript, by altering their input, e.g. leaving their input parameter blank returning the data of the other party which they wish to retrieve. ^159^

Kantarcioǧlu *et al.*, raise concerns about existing techniques for privacy-preserving data mining, such as adding noise to data or encryption-based approaches. ^160^ They emphasise the need to evaluate the privacy impact of data mining models. ^161^ They use a "medical diagnosis" scenario to illustrate the complexity of privacy issues, discussing the challenges of determining whether a classifier violates privacy when using public and private information. ^162^ The authors propose a framework for evaluating privacy loss in data mining results, offering precise definitions and formal analysis. ^163^ Section 2 presents a classification model, and Section 3 introduces a metric for privacy loss, providing examples and methods for calculation. ^164^ Their work aims to contribute to understanding and addressing privacy concerns in the context of data mining models. ^165^

In conclusion, literature examining the interplay between TDM, and data protection is sparse. There seems to be a focus on creating mining mechanisms which implement privacy by design and several scholars provide practical and technical solutions for achieving this. The common underlying message seems to be the manipulation of private information to avoid data breaches. Nevertheless there seems to be a correlation between data manipulation and data utility and a balance must be struck between the two to allow for privacy preserving TDM research to be carried out.

### TDM and fundamental rights

Another regulatory framework which may come into play when conducting TDM, which is sometimes examined by scholars, is that of fundamental rights. Some indirectly examine the interplay of TDM and fundamental rights, by exploring how TDM regulatory frameworks like those examined above, might interfere with fundamental rights whilst others, directly examine the act of TDM and how that might interact with fundamental rights. Several human rights may be at risk, depending on the type of TDM being carried out, and under which circumstances. This section aims to present the literature which examines the interplay between TDM and fundamental human rights.

***Indirect.*** A series of academic papers examine the recent EU CDSM TDM-specific exceptions, presented above, but under the guise of how such exceptions interact with fundamental human rights. For instance, Manteghi argues that the balancing mechanisms regulating their scope of application, may restrict one’s freedom of information and research enshrined under the freedom of expression. ^166^ Manteghi argues that the restriction of Article 3 of the CDSM to public sector entities, excluding private actors like journalists, individual researchers, and businesses raises concerns about restricting the freedom of information and research. ^167^ In an earlier paper, Manteghi argues that Article 3 and 4 of the CDSM which introduce the TDM-specific exceptions “ *are likely to create uncertainty about their coverage and scope, which possibly may undermine the users’ right to information”*. ^168^ Similarly, Geiger argues that the EU’s recent strategies for regulating AI resembles, a “ *football team that would be left without any strikers to score successfully and to win any of the competitions with other jurisdictions which may have the advantage of more flexible legal provisions allowing broader TDM activities*”. ^169^ Geiger argues that by restricting the scope of Article 3 of the CDSM, it may interfere with journalists’ freedom of expression highlighting the “Panama Papers” scandal as an example which may fall outside the scope of this new exception. ^170^ Geiger added that the Commission “ *risked creating an ineffective and therefore rapidly obsolete provision, in particular regarding the development of artificial intelligence, but also with regards to other activities of essential research and innovation not conducted by public bodies”*. ^171^ Scholars’ views regarding the narrow scope given to the EU TDM-specific exceptions were presented above, whereby their application is limited to content that is “lawfully accessed”. Manteghi argues that this concept should be interpreted in light of the term “lawful use” thereby ensuring that any harm caused by this scoping is proportional to the benefits that these exceptions are likely to provide in terms of copyright protection. ^172^ Furthermore, Synodinou argues that “lawful access” requires the adoption of a broad meaning within the confines of TDM extending to *“any use which is not restricted by law”. ^173^* Hence, any means by which lawful use has been obtained will inevitably allow for the application of those EU CDSM TDM-specific exceptions, arguably proportionally safeguarding one’s right to information and research. ^174^

***Direct.*** Tien, examining the US jurisdiction, takes a different approach towards examining the interplay between TDM and fundamental rights. ^175^ Tien considers the extensive information held by the US government on individuals, stored across various databases, raising concerns about civil liberties such as privacy, freedom of association, and freedom of speech questioning whether the Fourth Amendment, designed to protect against unreasonable searches and seizures, constrains government data mining activities. ^176^ “ *The Fourth Amendment's basic purpose is to safeguard the privacy and security of individuals against arbitrary invasions by governmental officials”*. ^177^ The author argues that the identification of patterns by mining across several databases, constitutes a search and thus cannot be knowingly exposed, and therefore, subjects of such searches deserve Fourth Amendment protection. ^178^

In summary, several scholars examine how the recent EU CDSM TDM-specific exceptions may interfere with one’s freedom of information and research, whilst another optic angle is provided by Tien who asserts that TDM itself might interfere with one’s right to privacy. As aforementioned, the interplay between TDM and fundamental rights is potentially multifaceted, depending on the type of TDM being carried out, and under which circumstances. For instance, one’s right to property which acts as the human rights foundation for the protection of intellectual property rights, enshrined in the EU under Article 17(2) of the Charter of Fundamental Rights, which states that “ *intellectual property rights shall be protected*”, may be interfered with when conducting TDM. Nevertheless, the CJEU clarified the scope of this right in stating that whilst “ *protection of the right to intellectual property is indeed enshrined in Article 17(2) of the Charter of Fundamental Rights of the European Union (‘the Charter’). There is, however, nothing whatsoever in the wording of that provision or in the Court’s case-law to suggest that that right is inviolable and must for that reason be absolutely protected”. ^179^* Hence, one’s right to intellectual property may be interfered with under certain circumstances. For this to happen, the CJEU requires a “ *balance to be struck between the various fundamental rights protected by the European Union”. ^180^* Thus, it can be concluded that another area of law which may come into play when conducting TDM is that of fundamental rights. The right interfered is dependent upon the type of TDM being carried out, and under which circumstances.

### TDM and contract law

So far, the arguments presented above, to a great extent, assume that no contract applies. It is, however, possible that contracts exist between hosts of databases and datasets and those users which want to access that data for mining purposes. Such contracts may restrict TDM from taking place. ^181^ For instance, many scientific publishers offer licenses which explicitly prohibit TDM from taking place. ^182^ Others apply their contracts through “terms of use” on their websites, which often also restrict TDM from taking place. ^183^ This section aims to present scholars’ views on the area where contract law and TDM meet.

The CDSM has arguably brought about changes in this intersection between contract law and TDM. ^184^ Above, author’s views regarding Article 4 of the CDSM were presented which arguably allows for rightsholders to opt-out from TDM activities to a certain extent restricting TDM through contracts. Nonetheless, Article 7(1) of the directive explicitly restricts contracts from overriding the mandatory exception found under Article 3 of the CDSM. ^185^ However, as discussed in previous sections and as pointed out by Meys, “ *in terms of material scope, the exception is limited to subject matter to which the beneficiaries have lawful access”,* which creates a contradiction as it conceptually implies that this exception will only apply to TDM that makes use of content that is freely available online or accessed based on “ *open access policies or contractual arrangements such as subscriptions*”. ^186^ Bearing in mind the discussion above, this lies on the opposite side of the legal spectrum when compared to its US counterpart where Carroll argued that in the US, users would, in theory be able to access, download and use works from Sci-Hub for the purposes of TDM. ^187^

Ducato and Strowel, examined the real-world impact contracts have on users who wish to conduct TDM. ^188^ The authors examined the terms and conditions of “ *21 online platforms, equally distributed among three sectors: mobility (carpooling and car sharing); accommodation (including services for sharing office space); and food (i.e. initiatives for the recuperation of unsold or unused food, sharing or delivery of home-cooked meals, etc.)*”. ^189^ They found that almost all (20 out of 21) websites examined either expressly or indirectly excluded TDM from taking place. ^190^ The authors assert that their analysis “ *shows a trend toward the general contractual ban of TDM*”. ^191^

McCracken and Raub who examined the difficulties researchers may face when conducting TDM in the US assert that “ *in some ways, TDM licensing still seems like a Wild West, where too many vendors are taking too many approaches, causing too many librarians to have to figure out far too many different licensing options*” *. ^192^* The authors attempted to establish “ *access for a collection of faculty and graduate students from a range of disciplines, departments, and even universities”. ^193^* When doing so they encountered significant and remarkable limitations in how much they were able to achieve with different content providers. ^194^

In a blog post Hugenholtz argues that the EU CDSM TDM-specific exceptions “ *effectively create and legitimise a derivative market for text and data mining, which right holders may wish to control, license or even entirely prohibit”. ^195^* Manteghi asserts that this may in turn “ *suppress the ability of users to access, share, or express protected content across the public and private spheres”*. ^196^ The author highlights the difficulties involved in obtaining rights clearance, which will indirectly benefit very large commercial companies or internet giants, whilst hindering start-ups and SMEs ability to progress in the field of AI. ^197^ As per Geiger “ *take it or leave it contractual conditions make access conditional upon accepting providers’ terms, while obtaining specific permission from various publishers to carry out TDM research can be extremely complex*”. ^198^ Manteghi highlights the need for Member States to deviate from this opt-out mechanism in a way in which is beneficial for start-ups and SMEs involved in AI development. ^199^

On the contrary, Senftleben argued that the opt-out mechanism for TDM under Article 4 of the CDSM could be transformed into frameworks that enhance the collection of descriptive and ownership data in favour of rightsholders. ^200^ They argue that these provisions provide an opportunity for the EU to enhance licensing opportunities for rightsholders. ^201^ If Europe fails to develop large-scale, low-cost licensing solutions, AI training may shift to regions with better infrastructure, risking significant revenue loss for the European creative industries. ^202^ Therefore, the creation of a comprehensive and interoperable data infrastructure is urgent to capitalize on these emerging opportunities. ^203^ Hence, unlike what other scholars have argued, where complex licensing agreements may ultimately stifle TDM research, Senftleben argues that Article 4 of the CDSM provides the opportunity to enhance metadata, streamlining licensing processes, allowing for rightsholders to more easily license their content for TDM purposes. On a similar note but using a different line of argument for a proposed solution, Tylec *et al.*, highlight the difficulties involved with identifying works used in training AI, and ensuring rightsholders are equitably remunerated. ^204^ They propose a solution similar to the private copying levy in the InfoSoc Directive. ^205^ With their proposed solution, AI developers would be allowed to use copyrighted works for training algorithms without needing individual licenses for each work. Instead, they would pay a statutory fee to compensate copyright holders, and the collected fees would be managed by collective management organisations. ^206^

In conclusion, contracts, and licensing agreements between hosts of datasets and those who wish to access said data for mining purposes, currently act as a barrier to free mining. To conduct lawful mining, users must navigate complex contractual terms and agreement procedures. Whilst some scholars, propose solutions for streamlining licensing agreements for TDM, there is still a gap in formal black letter law in this regard.

### TDM and competition law

Vesala proposes that one way in which these contractual hurdles for TDM may be addressed is through competition law. ^207^ This takes us onto the next section of this literature review which presents author’s views on the interplay between TDM and competition law. Vesala argues that EU competition law can assist in overcoming the obstacles created by contract law “ *by requiring copyright holders to provide access to works and grant licences for their use”. ^208^* Nevertheless, Vesala acknowledges the fact that such legal remedies offered by legal frameworks which regulate competition, may only be sought “ *when the practices concerned constitute abuse of a dominant position or a restrictive agreement, which is not automatically or even generally the case when access to or use of copyright protected materials is limited*”. ^209^

Beyond the remedies offered by EU competition law in overcoming contractual hurdles users conducting TDM may encounter, Kochelek examined the potential of using TDM to act anticompetitively, examining how such practices may come into conflict with US antitrust laws. ^210^ Kochelek argues that when data mining practices are employed against aggregated customer data, thereby enabling the identification of a consumers’ so-called “pain-point” i.e., the most undesirable value at which a party will still engage in a transaction, this in turn enables price discrimination. ^211^ The author ultimately argues that enforcing US antitrust rules against data-mining based price discrimination aligns with the US Sherman Act principles, which prescribe the rules governing free competition. ^212^

Hence, competition law may act as a bridge in overcoming contractual restrictions for users conducting TDM using datasets held by hosts which abuse their dominant position. Similarly, TDM may be used to enable anticompetitive practices.

### Generalist approach

Finally, some authors choose to take a generalist approach when examining “lawful TDM”. Such authors, highlight some of the key barriers standing between “free mining” and “lawful TDM”. This section aims to present such literature.

In a pre-Brexit era, Brook highlighted the EU regulatory framework governing TDM drawing examples from the UK’s approach. ^213^ The author stresses that copyright protection and the subsequent potential for copyright infringement acts as a barrier to users who wish to conduct TDM, examining the recent, at the time, introduction of a TDM-specific UK copyright exception for TDM carried out for the sole purpose of research for a non-commercial purpose, provided copying is accompanied by sufficient acknowledgement, except where this would be impossible. ^214^ The author nonetheless, argues that with the introduction of that exception “ *numerous constraints have, for UK researchers, been removed at a stroke”. ^215^* The author then highlights the difficulties database rights create for researchers wishing to conduct TDM. The author whilst only briefly describing the issues associated with database rights concludes that “ *as with copyright, the law here is often unclear and acts as a chilling effect and barrier to many”*. ^216^ Brook then moves onto discuss the issues created by complex licensing procedures highlighting the importance of creating open-access scientific literature through the use of creative commons licensing. ^217^ In an era preceding the CDSM, the author calls for a collective harmonised approach at EU level. ^218^ Hence, it can be deduced that Brook, in a pre-Brexit and CDSM era, highlighted some of the same difficulties that more recent scholarly work reviewed above, had also discussed.

In a 2014 study funded by the European Commission, when the UK was on the cusp of introducing their TDM specific exception, Triaille *et al.*, examined the copyright issues relevant to TDM in the EU in-depth, providing first a working definition of TDM and access to data choosing to use the term “data analysis” as an all-encompassing term. ^219^ They then analyse the exclusive rights afforded by copyright protection and how TDM might interfere with those, but also the potentially applicable exceptions at the time. ^220^ They subsequently examine other legal provisions that may be of relevance in the context of TDM, briefly highlighting issues relating to data protection, contract law and unfair competition. ^221^ They conclude by suggesting the introduction of a “new “data analysis exception” applying solely to TDM, which greatly resembles the CDSM exceptions. ^222^

Truyens and Van Eecke provide an analysis of the regulatory frameworks applicable to text mining, distinguishing it from data mining. ^223^ The authors examine how text mining interacts with copyright law analysing the criteria for assessing originality and the applicability of exceptions. ^224^ The authors then describe how text mining might come into conflicts with database rights, and contract law, ^225^ before providing their assessment claiming that a legal issue within the context of text-mining is fact-dependant and that “ *only for some delineated areas (very old texts, legal statutes, texts in the public domain) strong legal certainty can be obtained without case-by-case assessments*”. ^226^ The authors suggest that rigid legal rules in the EU might hinder scientific research and technological developments related to text mining. ^227^

Guadamuz and Cabel, focus on an analysis of content mining in research institutions and the challenges faced by both users and creators and as aforementioned, claim that legal issues arise at the point at which a user conducting TDM obtains access to their dataset and apply mining tools to said dataset. ^228^ They too focus on an analysis of copyright law, database rights and licensing, calling for an increase in open access policies enabling greater access to mining. ^229^

Meys, examines the existing limitations on data reusability imposed by database rights and contracts, whilst analysing the EU CDSM TDM-specific exceptions. ^230^ The author ultimately concludes that copyright, sui generis database rights and contracts pose obstacles to data re-usability for AI development. ^231^

Rosati who as mentioned in the first section of this review, identifies three general TDM processes “ *1) Access to content, 2) Extraction and/or copying of content, and 3) Mining of text and/or data for knowledge discovery*” each of which with their own legal consideration, also adopts a generalist approach highlighting several areas of law that may make up the regulatory framework governing TDM. ^232^ Namely, when analysing the first TDM process – “access to content” – Rosati discusses contractual issues that may arise in terms of obtaining a license to mine. ^233^ The added difficulties that may arise when dealing with orphan works and out-of-commerce works are also discussed. ^234^ When examining the second TDM process – “ *extraction and/or copying of content”* – Rosati highlights the legal concerns that may arise within the context of copyright and sui generis (database) rights. ^235^ The author then finally notes that other areas of law may be relevant at this stage such as data protection/privacy laws, and contract law. ^236^ In the third stage of TDM – “ *mining of text and/or data and knowledge discovery”* – the author provides a procedural analysis of what a typical TDM activity would involve, in terms of knowledge discovery. ^237^ Upon analysing these TDM processes, Rosati then moves onto a jurisdictional comparison of “ *jurisdictions that display a more frequent and advanced use of TDM”*, examining the US’s fair use, the UK’s TDM exception and the EU’s CDSM TDM-specific exceptions. ^238^ Rosati concludes by stating that as EU Member States transpose the CDSM into their laws, caution is urged to ensure that EU businesses engaging in TDM are not disadvantaged compared to non-EU counterparts, considering TDM's potential impact on AI and AI-generated creativity. ^239^

Hammon *et al.* discuss the rapid growth of AI applications particularly since the release of ChatGPT in November 2022, emphasising the need for businesses to consider various legal and compliance risks associated with the development of AI. ^240^ The authors provide an overview of the regulatory frameworks such businesses are likely to encounter, such as contractual protection and AI specific regulations such as the EU’s AI Act discussed above. ^241^ They then separate their analysis into input and output risks, examining regulatory risks associated with what the AI is fed as input and what output an AI is likely to produce respectively. ^242^ In terms of input risks the authors highlight that there are likely data protection and intellectual property risks. ^243^ Similarly with regards to output risks the authors highlight the intellectual property risks associated. ^244^ They then examine what they call “overarching considerations”, highlighting the environmental risks related to the energy consumption of AI systems, social risks related to the takeover of a business’s workforce by AI, and the importance of ethical policies, audits, and alignment with corporate values in AI adoption is emphasised. ^245^

Finally, in a recent paper, Sean M Fill-Flynn summarises the key points that have emerged out of the wider project on the Right to Research in International Copyright. First, the author highlights the importance of TDM which involves analysing digitized information and is crucial for various AI tools, including generative AI models whilst it is also widely used in academic research across different fields. ^246^ Second, the importance of TDM is emphasised, demonstrating how it is linked to fundamental rights like receiving and imparting information and benefiting from culture and science, often referred to as the “ *right to research*”. ^247^ Finally, the author exemplifies the barriers that copyright created for TDM research. First, a patchwork of frameworks across multiple jurisdictions create barriers for TDM research especially in cross-border projects. ^248^ Second, licensing models for regulating access to text and data hinder research and economic activity, particularly affecting resource-dependent researchers and AI industries, especially in developing countries. ^249^ Open research exceptions could benefit TDM research, and claims of harm to creators from open exceptions lack empirical support. ^250^ Finally, there is a trend towards open exceptions especially for research purposes and the right to research should be advocated for in international copyright law. ^251^

## Conclusion

TDM is here to stay. It seems that the regulatory framework is a game of “cat and mouse” where the existing system of laws not always specifically attuned towards regulating TDM continue to apply. Gaps in existing black letter law are iteratively filled by new attempts to regulate the realm of TDM. The recent introduction of TDM-specific exceptions at EU level, although criticised, is a welcomed advancement in the field of regulating TDM in a way in which balances stakeholders’ interests. Nonetheless, there is still a way to go in terms of holistically regulating TDM.

Currently, users who conduct TDM must navigate this complex web of legal rules without a map. This research as a whole, which this literature review forms part of, aims to create a practical guide and tool facilitating lawful TDM.

What this literature review makes evident is that literature examining “lawful TDM” is fragmented. Whilst some attempt to provide a more general interpretation of how one can conduct “lawful TDM”, this literature review illuminates the fact that they are not all-encompassing. The regulatory frameworks that apply to users conducting TDM are multifaceted, and ever-changing on a case-by-case basis, which heavily depends upon the type of mining being conducted, where mining takes place, the dataset involved, how such data is accessed, and how the output of mining processes will ultimately be used. There is an ever-growing need for a holistic interpretation of the regulatory frameworks which apply, creating a map which would allow for users conducting TDM to navigate this complex web of legal rules.

## Data Availability

No data are associated with this article Zenodo: Appendix for Review Paper Entitled: “A literature review of “lawful” text and data mining.” ( https://doi.org/10.5281/zenodo.12654691) (Vrakas, 2024). ^252^ The project contains the following extended data: A Literature Review of Lawful Text and Data Mining - Appendix.pdf Data is available under the terms of the Creative Commons Attribution 4.0 International License (CC-BY 4.0).

## Footnotes

^1^ Melvin M Vopson, ‘The World’s Data Explained: How Much We’re Producing and Where It’s All Stored’ ( *The Conversation*) < http://theconversation.com/the-worlds-data-explained-how-much-were-producing-and-where-its-all-stored-159964> accessed 23 June 2022.

^2^ ‘Total Data Volume Worldwide 2010-2025’ ( *Statista*) < https://www.statista.com/statistics/871513/worldwide-data-created/> accessed 23 June 2022.

^3^ European Commission, ‘European Data Market Study (SMART 2013/0063)’ < https://digital-strategy.ec.europa.eu/en/library/european-data-market-study-update> accessed 24 June 2022.

^4^ Peter C Verhoef and others, ‘Digital Transformation: A Multidisciplinary Reflection and Research Agenda’ (2021) 122 Journal of Business Research 889.

^5^ Luisa Gatti, ‘The European Solution for Text and Data Mining: A Focus on the Opt-Out System Provided by Article 4 of the DSM Directive’ (2021) 43 European Intellectual Property Review 765.

^6^ James Caudwell, ‘LibGuides: Text & Data Mining: What Is TDM?’ < https://libguides.cam.ac.uk/tdm/definitions> accessed 7 November 2023; ‘Text and Data Mining at Springer Nature’ < https://www.springernature.com/gp/researchers/text-and-data-mining#:~:text=TDM%20(Text%20and%20Data%20Mining,can%20provide%20valuable%20information%20needed> accessed 7 November 2023.

^7^ Caudwell (n 6); Marti A Hearst, ‘Untangling Text Data Mining’, *Proceedings of the 37th annual meeting of the Association for Computational Linguistics on Computational Linguistics* (Association for Computational Linguistics 1999) < https://dl.acm.org/doi/10.3115/1034678.1034679> accessed 18 January 2024; Sonali VijayGaikwad, Archana Chaugule and Pramod Patil, ‘Text Mining Methods and Techniques’ (2013) 85 International Journal of Computer Applications.

^8^ Caudwell (n 6).

^9^ Thilla Rajaretnam, ‘Data Mining and Data Matching: Regulatory and Ethical Considerations Relating to Privacy and Confidentiality in Medical Data’ (2014) 9 Journal of International Commercial Law and Technology 294.

^10^ Douglas M Kochelek, ‘Data Mining and Antitrust’ (2009) 22 Harvard Journal of Law & Technology 515.

^11^ Caudwell (n 6); ‘Text and Data Mining at Springer Nature’ (n 6).

^12^ Marinos Papadopoulos and others, ‘Empirical Research on Web Harvesting in the Process of Text and Data Mining in National Libraries of EU Member States’ (2019) 10 Open Journal of Philosophy 88; Liane Colonna, ‘Opportunities and Challenges to Utilizing Text-Data Mining in Public Libraries : A Need for Legal Research’ (2018) 65 Scandinavian Studies in Law 191.

^13^ Nicolas Jondet, ‘The Text and Data Mining Exception in the Proposal for a Directive on Copyright: Why the European Union Needs to Go Further than the Laws of Member States.’ (2018) 67 Propriétés Intellectuelles 25; Eleonora Rosati, ‘Copyright as an Obstacle or an Enabler? A European Perspective on Text and Data Mining and Its Role in the Development of AI Creativity’ (2020) 27 Asia Pacific Law Review 1.

^14^ Rajaretnam (n 9); Sean M Fiil-Flynn and others, ‘Legal Reform to Enhance Global Text and Data Mining Research’ (2022) 378 Science 951.

^15^ Matías Jackson Bertón, ‘Text and Data Mining Exception in South America: A Way to Foster AI Development in the Region’ (2021) 70 GRUR International 1145; C Handke, L Guibault and JJ Vallbé, *Is Europe Falling Behind in Data Mining? Copyright’s Impact on Data Mining in Academic Research* (AmsterdamIOS Press 2015) < https://dare.uva.nl/search?identifier=1b29a7cf-aa67-47d9-80c7-b2fd790ffd87> accessed 8 November 2023.

^16^ Adam Wyner and others, ‘Approaches to Text Mining Arguments from Legal Cases’ (2010).

^17^ Thomas Margoni, ‘Text and Data Mining in Intellectual Property Law: Towards an Autonomous Classification of Computational Legal Methods’ [2020] CREATe < https://www.create.ac.uk/blog/2020/05/01/new-working-paper-text-and-data-mining-in-intellectual-property-law-towards-an-autonomous-classification-of-computational-legal-methods/> accessed 1 December 2023.

^18^ Maarten Truyens and Patrick Van Eecke, ‘Legal Aspects of Text Mining’ in Nicoletta Calzolari and others (eds), *Proceedings of the Ninth International Conference on Language Resources and Evaluation (LREC’14)* (European Language Resources Association (ELRA) 2014) < http://www.lrec-conf.org/proceedings/lrec2014/pdf/452_Paper.pdf> accessed 8 November 2023; Matthew Sag, ‘The New Legal Landscape for Text Mining and Machine Learning’ (2019) 66 Journal of the Copyright Society of the U.S.A. 291.

^19^ Caudwell (n 6); VijayGaikwad, Chaugule and Patil (n 7).

^20^ Liane Colonna, ‘A Taxonomy and Classification of Data Mining’ (2017) 16 SMU Science and Technology Law Review 309.

^21^ Andrea Katalin Tóth, ‘Algorithmic Copyright Enforcement and AI: Issues and Potential Solutions through the Lens of Text and Data Mining’ (2019) 13 Masaryk University Journal of Law and Technology 361, 378.

^22^ Michael W Carroll, *Copyright and the Progress of Science: Why Text and Data Mining Is Lawful* (UC Davis School of Law 2019) < https://papers.ssrn.com/sol3/papers.cfm?abstract_id=3531231> accessed 8 November 2023.

^23^ Fiil-Flynn and others (n 14).

^24^ Rosati (n 13).

^25^ Andrés Guadamuz and Diane Cabell, ‘Data Mining in UK Higher Education Institutions: Law and Policy’ (2014) 4 Queen Mary Journal of Intellectual Property 3, 5.

^26^ ibid 5–6.

^27^ ibid 6.

^28^ Sean Flynn and others, ‘Implementing User Rights for Research in the Field of Artificial Intelligence: A Call for International Action’ [2020] Joint PIJIP/TLS Research Paper Series < https://digitalcommons.wcl.american.edu/research/48>.

^29^ See for instance, Pamela Samuelson, ‘Text and Data Mining of In-Copyright Works: Is It Legal?’ (2021) 64 Communications of the ACM 20; Jackson Bertón (n 15).

^30^ Fiil-Flynn and others (n 14).

^31^ Directive 2001/29 on the harmonisation of certain aspects of copyright and related rights in the information society [2001] OJ L167/10.

^32^ Directive 2009/24 on the legal protection of computer programs [2009] OJ L111/16.

^33^ Directive 96/9 on the legal protection of databases [1996] OJ L77/20.

^34^ See for instance, Christophe Geiger, Giancarlo Frosio and Oleksandr Bulayenko, ‘Text and Data Mining in the Proposed Copyright Reform: Making the EU Ready for an Age of Big Data?’ (2018) 49 IIC - International Review of Intellectual Property and Competition Law 814, 817; Martin Kretschmer and Thomas Margoni, ‘Data Mining: Why the EU’s Proposed Copyright Measures Get It Wrong’; Thomas Margoni and Martin Kretschmer, ‘A Deeper Look into the EU Text and Data Mining Exceptions: Harmonisation, Data Ownership, and the Future of Technology’ (2022) 71 GRUR International 685; Reto Hilty and Heiko Richter, ‘Position Statement of the Max Planck Institute for Innovation and Competition on the Proposed Modernisation of European Copyright Rules Part B Exceptions and Limitations (Art. 3 – Text and Data Mining)’ (Max Planck Institute for Innovation & Competition 2017) Research Paper No. 17-02 para 3; Jonathan Griffiths, Tatiana Synodinou and Raquel Xalabarder, ‘Comment of the European Copyright Society Addressing Selected Aspects of the Implementation of Articles 3 to 7 of Directive (EU) 2019/790 on Copyright in the Digital Single Market’ (2023) 72 GRUR International 22.

^35^ Directive (EU) 2019/790, Recital 9.

^36^ See CJEU, C-5/08, *Infopaq International A/S v. Danske Dagblades Forening* (16 July 2009). ECLI:EU:C:2009:465, paras. 54–55 (finding that even an excerpt of 11 words might be protected).

^37^ Signatories to the Berne Convention, which include all but 20 countries of the world will grant rightsholders the right to reproduce their work. See Berne Convention for the Protection of Literary and Artistic Works (as amended on September 28, 1979), Article 9(1). In the EU this right is enshrined under Article 2 of Directive 2001/29.

^38^ Milad Nasr and others, ‘Scalable Extraction of Training Data from (Production) Language Models’ (arXiv, 28 November 2023) < http://arxiv.org/abs/2311.17035> accessed 4 December 2023.

^39^ Antonios Baris, ‘AI Covers: Legal Notes on Audio Mining and Voice Cloning’ [2024] Journal of Intellectual Property Law & Practice jpae029, 3.

^40^ Martin Senftleben, ‘Compliance of National TDM Rules with International Copyright Law: An Overrated Nonissue?’ (2022) 53 IIC - International Review of Intellectual Property and Competition Law 1477.

^41^ Directive 96/9 on the legal protection of databases [1996] OJ L77/20.

^42^ Rosati (n 13).

^43^ Directive (EU) 2019/790 of the European Parliament and of the Council of 17 April 2019 on copyright and related rights in the Digital Single Market and amending Directives 96/9/EC and 2001/29/EC.

^44^ Regulation 3 of the Copyright and Rights in Performances (Research, Education, Libraries and Archives) Regulations 2014, No. 1372, adding Art. 29A to the Copyright, Designs and Patents Act 1988.

^45^ Copyright, Designs and Patents Act 1988, section 29A.

^46^ Art. 38 of Law No. 2016-1231 for a Digital Republic added paragraph 10 to Art. L122-5 and paragraph 5 to Art. L342-3 of the Intellectual Property Code ( *Code de la proprie´te´ intellectuelle*) (CPI).

^47^ Estonian Copyright Act, Art. 19(3), and Urheberrechtsgesetz § 60d respectively.

^48^ For a discussion of this, see Maria Kanellopoulou - Botti and others, ‘Legal and Technical Issues for Text and Data Mining in Greece’ (2019) 2019 Computer Ethics - Philosophical Enquiry (CEPE) Proceedings < https://digitalcommons.odu.edu/cepe_proceedings/vol2019/iss1/11>.

^49^ Geiger, Frosio and Bulayenko (n 34) 830.

^50^ Jackson Bertón (n 15).

^51^ Jondet (n 13); Kretschmer and Margoni (n 34).

^52^ Article 3 of Directive (EU) 2019/790.

^53^ Article 4 of Directive (EU) 2019/790.

^54^ See for instance, Μaria Bottis and others, ‘Text and Data Mining in Directive 2019/790/EU Enhancing Web-Harvesting and Web-Archiving in Libraries and Archives’ (2019) 09 Open Journal of Philosophy 369; Tóth (n 21).

^55^ Papadopoulos and others (n 12).

^56^ Rossana Ducato and Alain Strowel, ‘Ensuring Text and Data Mining: Remaining Issues with the EU Copyright Exceptions and Possible Ways Out’ (2021) 43 European Intellectual Property Review 322, 331.

^57^ Ducato and Strowel (n 56).

^58^ Recital 14 of Directive (EU) 2019/790.

^59^ Directive (EU) 2019/790, Article 7(1) states that “ *any contractual provision contrary to the exceptions provided for in Articles 3 […] shall be unenforceable*.” See also, Gatti (n 5) 767.

^60^ Gatti (n 5); Christophe Geiger, Giancarlo Frosio and Oleksandr Bulayenko, ‘Text and Data Mining: Articles 3 and 4 of the Directive 2019/790/EU’ (2019); Nicolas Binctin, ‘TDM: A CHALLENGE FOR ARTIFICIAL INTELLIGENCE’ [2019] Revue In‐ternationale du Droit d’Auteur.

^61^ Griffiths, Synodinou and Xalabarder (n 34) 11–12.

^62^ ibid 12; Margoni and Kretschmer (n 34).

^63^ Geiger, Frosio and Bulayenko (n 60).

^64^ Paul Keller, ‘Generative AI and Copyright: Convergence of Opt-Outs?’ ( *Kluwer Copyright Blog*, 23 November 2023) < https://copyrightblog.kluweriplaw.com/2023/11/23/generative-ai-and-copyright-convergence-of-opt-outs/> accessed 27 November 2023.

^65^ ibid.

^66^ ibid.

^67^ Martin Senftleben, ‘AI Act and Author Remuneration - A Model for Other Regions?’ (24 February 2024) < https://papers.ssrn.com/abstract=4740268> accessed 29 May 2024.

^68^ ibid 1.

^69^ ibid 4.

^70^ Article 6, Directive 2001/29 on the harmonisation of certain aspects of copyright and related rights in the information society [2001] OJ L167/10.

^71^ Andrée Glancia MADINDA, ‘THE TEXT AND DATA MINING EXCEPTION IN THE DIRECTIVE ON COPYRIGHT IN THE DIGITAL SINGLE MARKET: A STORMY OUTLOOK FOR AUTHOR’S RIGHTS’ [2022] Winner of the 2022 edition of the GESAC/ALAI European Authors’ Right Award.

^72^ ‘Europe’s TDM Exception for Research: Will It Be Undermined By Technical Blocking From Publishers?’ ( *LIBER Europe*) < https://libereurope.eu/article/tdm-technical-protection-measures/> accessed 7 February 2024.

^73^ Hilty and Richter (n 34) para 29.

^74^ Stepanka Havlikova, ‘Web Scraping and Text and Data Mining Exception: Could the Cdsm Directive, Designed to Support the Reuse of Publicly Available Data, Have Had the Opposite Effect?’ (18 October 2023) < https://papers.ssrn.com/abstract=4605551> accessed 29 May 2024.

^75^ ibid 14.

^76^ ibid 14–15.

^77^ ibid 17.

^78^ ibid.

^79^ ibid 24.

^80^ Geiger, Frosio and Bulayenko (n 60).

^81^ Rosati (n 13).

^82^ Maryna Manteghi, ‘Overcoming Barriers to Text and Data Mining in the Era of ChatGPT: The Proposed Data Act as a Game-Changer’ [2023] GRUR International.

^83^ ibid.

^84^ Article 5(1) of Directive 2001/29. See Margoni and Kretschmer (n 34) 691.

^85^ ibid 694.

^86^ Alexandre L Dias Pereira, ‘TDM Copyright for AI in Europe: A View from Portugal’ (2023) 18 Journal of Intellectual Property Law & Practice 900.

^87^ Micke Lindholm, ‘Text and Data Mining under Finnish Copyright Law before and after the DSM Directive’ (2020) < https://helda.helsinki.fi/items/42a78c1d-da42-4461-a7f1-37b66c4d3cb6> accessed 14 November 2023.

^88^ Bernardo Calabrese, ‘Scientific TDM Exception and Communication to the Public: Did Italians Do It Better … or at Least Not Worse?’ (2022) 17 Journal of Intellectual Property Law & Practice 399.

^89^ ibid.

^90^ Samuelson (n 29).

^91^ Artha Dermawan, ‘Text and Data Mining Exceptions in the Development of Generative AI Models: What the EU Member States Could Learn from the Japanese “Nonenjoyment” Purposes?’ The Journal of World Intellectual Property < https://onlinelibrary.wiley.com/doi/abs/10.1111/jwip.12285> accessed 18 January 2024.

^92^ Eleonora Rosati, ‘No Step-Free Copyright Exceptions: The Role of the Three-Step in Defining Permitted Uses of Protected Content (Including TDM for AI-Training Purposes)’ (2023) 123.

^93^ Paul Keller, ‘TDM: Poland Challenges the Rule of EU Copyright Law’ ( *Kluwer Copyright Blog*, 20 February 2024) < https://copyrightblog.kluweriplaw.com/2024/02/20/tdm-poland-challenges-the-rule-of-eu-copyright-law/> accessed 21 February 2024.

^94^ ibid.

^95^ Stéphan André, ‘The Legal Evolution of the Text and Data Mining Copyright Exceptions in the European Union - From Fragmentation to Uniformity?’ (2022) 63.

^96^ Carroll (n 22); Edward Lee, ‘Technological Fair Use’ (2010) 83 Southern California Law Review 797; Jerome Reichman and Ruth Okediji, ‘When Copyright Law and Science Collide: Empowering Digitally Integrated Research Methods on a Global Scale’ (2012) 96 Minnesota Law Review 1362; Matthew Sag, ‘Copyright and Copy-Reliant Technology.’ [2009] Faculty Publications & Other Works.

^97^ Authors Guild, Inc. v. Google, Inc., 804 F.3d 202 (2d Cir. 2015). See for instance, Benjamin Goh, ‘Two Ways of Looking at a Printed Book’ (2022) 85 The Modern Law Review 697.

^98^ Authors Guild, Inc. v. HathiTrust, 755 F.3d 87 (2d Cir. 2014).

^99^ Sag (n 18) 293.

^100^ ibid 302.

^101^ ibid.

^102^ ibid 301–302.

^103^ ibid 320.

^104^ Carroll (n 22) 954.

^105^ Pamela Samuelson, ‘A Bold Take on Copyright Implications of Text & Data Mining’ [2021] JOTWELL < https://ip.jotwell.com/a-bold-take-on-copyright-implications-of-text-data-mining/> accessed 10 November 2023.

^106^ Thomas Margoni, ‘Saving Research: Lawful Access to Unlawful Sources under Art. 3 CDSM Directive?’ ( *Kluwer Copyright Blog*, 22 December 2023) < https://copyrightblog.kluweriplaw.com/2023/12/22/saving-research-lawful-access-to-unlawful-sources-under-art-3-cdsm-directive/> accessed 18 January 2024.

^107^ ibid.

^108^ U.S. Code § 1201.

^109^ Quinn Dombrowski and Lauren Tilton, ‘Access and Advocacy: Text & Data Mining and DMCA §1201’ (2024) 13 Digital Studies / Le champ numérique < https://www.digitalstudies.org/article/id/9658/> accessed 29 May 2024.

^110^ ibid 12.

^111^ ibid 14.

^112^ Sean Flynn and others, ‘Research Exceptions in Comparative Copyright’ [2022] Joint PIJIP/TLS Research Paper Series < https://digitalcommons.wcl.american.edu/research/75>.

^113^ ibid 17.

^114^ ibid 36.

^115^ ibid 37.

^116^ Rosati (n 13).

^117^ Handke, Guibault and Vallbé (n 15).

^118^ ibid 18.

^119^ Martin Kretschmer, Thomas Margoni and Pinar Oruç, ‘Copyright Law and the Lifecycle of Machine Learning Models’ (2024) 55 IIC - International Review of Intellectual Property and Competition Law 110.

^120^ Lita van Wel and Lambèr Royakkers, ‘Ethical Issues in Web Data Mining’ (2004) 6 Ethics and Information Technology 129.

^121^ Colonna (n 12).

^122^ Pattaraporn Sangaroonsilp and others, ‘Mining and Classifying Privacy and Data Protection Requirements in Issue Reports’ (arXiv, 2015) < http://arxiv.org/abs/2112.13994> accessed 21 November 2023.

^123^ Edward S Dove, ‘The EU General Data Protection Regulation: Implications for International Scientific Research in the Digital Era’ (2018) 46 The Journal of Law, Medicine & Ethics 1013, 1013.

^124^ Colonna (n 12).

^125^ Dove (n 123).

^126^ Rashid Zaman and Marwan Hassani, ‘Process Mining Meets GDPR Compliance: The Right to Be Forgotten as a Use Case’ [2019] ICPM Doctoral Consortium 2019 < http://www.scopus.com/inward/record.url?scp=85071764645&partnerID=8YFLogxK> accessed 22 November 2023.

^127^ ibid.

^128^ Nils Gruschka and others, ‘Privacy Issues and Data Protection in Big Data: A Case Study Analysis under GDPR’, *2018 IEEE International Conference on Big Data (Big Data)* (2018) < https://ieeexplore.ieee.org/document/8622621> accessed 21 November 2023.

^129^ Article 83(40, EU General Data Protection Regulation (GDPR): Regulation (EU) 2016/679 of the European Parliament and of the Council of 27 April 2016 on the protection of natural persons with regard to the processing of personal data and on the free movement of such data, and repealing Directive 95/46/EC (General Data Protection Regulation), OJ 2016 L 119/1.

^130^ Article 25, EU General Data Protection Regulation (GDPR): Regulation (EU) 2016/679 of the European Parliament and of the Council of 27 April 2016 on the protection of natural persons with regard to the processing of personal data and on the free movement of such data, and repealing Directive 95/46/EC (General Data Protection Regulation), OJ 2016 L 119/1.

^131^ Gruschka and others (n 128); Daniel Solove, ‘Why I Love the GDPR: 10 Reasons’ ( *TeachPrivacy*, 2 May 2018) < https://teachprivacy.com/why-i-love-the-gdpr/> accessed 21 November 2023.

^132^ Sangaroonsilp and others (n 122).

^133^ ibid.

^134^ ibid.

^135^ Chris Clifton and Don Marks, ‘Security and Privacy Implications of Data Mining’ [2000] ACM SIGMOD Workshop on Research Issues on Data Mining and Knowledge Discovery.

^136^ ibid.

^137^ Bart W Schermer, ‘The Limits of Privacy in Automated Profiling and Data Mining’ (2011) 27 Computer Law & Security Review 45.

^138^ ibid.

^139^ ibid 48.

^140^ ibid 52.

^141^ ibid 50.

^142^ ibid.

^143^ Chris Clifton, Murat Kantarcioglu and Jaideep Vaidya, ‘Defining Privacy for Data Mining’ (2003).

^144^ ibid 198.

^145^ ibid.

^146^ ibid.

^147^ ibid 199.

^148^ ibid 200.

^149^ ibid.

^150^ ibid.

^151^ ibid 200–201.

^152^ ibid 202.

^153^ Dakshi Agrawal and Charu C Aggarwal, ‘On the Design and Quantification of Privacy Preserving Data Mining Algorithms’, *Proceedings of the twentieth ACM SIGMOD-SIGACT-SIGART symposium on Principles of database systems* (Association for Computing Machinery 2001) < https://dl.acm.org/doi/10.1145/375551.375602> accessed 23 November 2023.

^154^ ibid.

^155^ Jaideep Vaidya, Yu Michael Zhu and Chris Clifton, *Privacy Preserving Data Mining*, vol 19 (Springer US 2006) < http://link.springer.com/10.1007/978-0-387-29489-6> accessed 23 November 2023.

^156^ Lindell and Pinkas, ‘Privacy Preserving Data Mining’ (2002) 15 Journal of Cryptology 177.

^157^ ibid.

^158^ ibid.

^159^ ibid 2.

^160^ Murat Kantarcioǧlu, Jiashun Jin and Chris Clifton, ‘When Do Data Mining Results Violate Privacy?’, *Proceedings of the tenth ACM SIGKDD international conference on Knowledge discovery and data mining* (Association for Computing Machinery 2004) < https://dl.acm.org/doi/10.1145/1014052.1014126> accessed 23 November 2023.

^161^ ibid.

^162^ ibid.

^163^ ibid.

^164^ ibid.

^165^ ibid.

^166^ Maryna Manteghi, ‘In Search of Balance: Text, Data Mining and Copyright in the Digital Single Market Directive from a Fundamental Rights Perspective - UTU Research Portal - UTU Research Portal’ (2023) 48 European Law Review 443.

^167^ ibid 448–449.

^168^ Maryna Manteghi, ‘Text and Data Mining in the EU: Managing a Conflict Between Copyright and the Right to Information’ (2021) 43 European Intellectual Property Review 701.

^169^ Christophe Geiger, ‘The Missing Goal-Scorers in the Artificial Intelligence Team: Of Big Data, the Fundamental Right to Research and the Failed Text and Data Mining Limitations in the CSDM Directive’ [2021] Joint PIJIP/TLS Research Paper Series 5.

^170^ ibid 6–7.

^171^ ibid 7.

^172^ Manteghi, ‘In Search of Balance: Text, Data Mining and Copyright in the Digital Single Market Directive from a Fundamental Rights Perspective - UTU Research Portal - UTU Research Portal’ (n 166) 452.

^173^ Tatiana Eleni Synodinou, ‘Lawfulness for Users in European Copyright Law: Acquis and Perspectives’ (2019) 10 JIPITEC para 20 < https://www.jipitec.eu/issues/jipitec-10-1-2019/4876>.

^174^ Manteghi, ‘In Search of Balance: Text, Data Mining and Copyright in the Digital Single Market Directive from a Fundamental Rights Perspective - UTU Research Portal - UTU Research Portal’ (n 166) 453.

^175^ Lee Tien, ‘Privacy, Technology and Data Mining’ (2004) 30 Ohio Northern University Law Review 389.

^176^ ibid.

^177^ ibid 400.

^178^ ibid 408–415.

^179^ C-70/10. *Scarlet Extended v. SABAM,* [2011] ECR I-11959 para. 43.

^180^ C-469/17 *Funke Medien NRW GmbH v. Bundesrepublik Deutschland,* ECLI:EU:C:2019:623, para. 53.

^181^ See for instance, Rosati (n 11) 12 who discusses that contracts may restrict TDM at the point that she calls the second step of TDM: Extraction and/or copying of content or Dorian Cougias and others, *Data Protections and Licenses Affecting Text and Data Mining for Machine Learning* (2022) who argue that the complex interplay of copyright and the resulting need to license unreasonably restrict TDM in the research context.

^182^ Truyens and Van Eecke (n 18).

^183^ ibid.

^184^ Romain Meys, ‘Data Mining Under the Directive on Copyright and Related Rights in the Digital Single Market: Are European Database Protection Rules Still Threatening the Development of Artificial Intelligence?’ (2020) 69 GRUR International 457.

^185^ Directive (EU) 2019/790, Article 7(1).

^186^ Meys (n 184) 465.

^187^ Carroll (n 22) 954–959. See section “1. TDM and Intellectual Property Rights”.

^188^ Rossana Ducato and Alain Strowel, ‘Limitations to Text and Data Mining and Consumer Empowerment: Making the Case for a Right to “Machine Legibility”’ (2019) 50 IIC - International Review of Intellectual Property and Competition Law 649.

^189^ ibid 668.

^190^ ibid 674.

^191^ ibid.

^192^ Peter McCracken and Emma Raub, ‘Licensing Challenges Associated With Text and Data Mining: How Do We Get Our Patrons What They Need?’ (2023) 11 Journal of Librarianship and Scholarly Communication 13 < https://www.iastatedigitalpress.com/jlsc/article/id/15530/> accessed 30 November 2023.

^193^ ibid 3.

^194^ ibid.

^195^ P Bernt Hugenholtz, ‘The New Copyright Directive: Text and Data Mining (Articles 3 and 4)’ ( *Kluwer Copyright Blog*, 24 July 2019) < https://copyrightblog.kluweriplaw.com/2019/07/24/the-new-copyright-directive-text-and-data-mining-articles-3-and-4/> accessed 28 November 2023.

^196^ Maryna Manteghi, ‘The Insufficiency of the EU’s Text and Data Mining Exceptions for Using Artificial Intelligence’ [2022] European Intellectual Property Review 660–661 < https://www.westlaw.com/Document/I08AA9B604C1B11EDBF0AD27084713A21/View/FullText.html?transitionType=Default&contextData=(sc.Default)&VR=3.0&RS=cblt1.0&sp=wlnintell-000> accessed 28 November 2023.

^197^ ibid 661.

^198^ Geiger, Frosio and Bulayenko (n 34) 826.

^199^ Manteghi, ‘The Insufficiency of the EU’s Text and Data Mining Exceptions for Using Artificial Intelligence’ (n 196) 661.

^200^ Martin Senftleben, ‘Copyright Data Improvement for AI Licensing – The Role of Content Moderation and Text and Data Mining Rules’ (4 May 2024) < https://papers.ssrn.com/abstract=4817796> accessed 29 May 2024.

^201^ ibid 6–8.

^202^ ibid.

^203^ ibid.

^204^ Grzegorz Tylec and others, ‘Is It Possible to License Works Used in the Learning Process of Artificial Intelligence Algorithms?’ (16 February 2024) < https://papers.ssrn.com/abstract=4729495> accessed 29 May 2024.

^205^ ibid.

^206^ ibid 12–13.

^207^ Juha Vesala, ‘Developing Artificial Intelligence-Based Content Creation: Are EU Copyright and Antitrust Law Fit for Purpose?’ (2023) 54 IIC - International Review of Intellectual Property and Competition Law 351.

^208^ ibid 368.

^209^ ibid.

^210^ Kochelek (n 10).

^211^ ibid 520–523.

^212^ ibid 526–534.

^213^ Michelle Brook, Peter Murray-Rust and Charles Oppenheim, ‘The Social, Political and Legal Aspects of Text and Data Mining (TDM)’ (2014) 20 D-Lib Magazine < http://www.dlib.org/dlib/november14/brook/11brook.html> accessed 30 November 2023.

^214^ ibid.

^215^ ibid.

^216^ ibid.

^217^ ibid.

^218^ ibid.

^219^ De Wolf & Partners and others, *Study on the Legal Framework of Text and Data Mining (TDM)* (Publications Office of the European Union 2014) < https://data.europa.eu/doi/10.2780/1475> accessed 27 June 2022.

^220^ ibid 28–84.

^221^ ibid 89–93.

^222^ ibid 116–117.

^223^ Truyens and Van Eecke (n 18).

^224^ ibid 2182–2183.

^225^ ibid 2184–2185.

^226^ ibid 2185.

^227^ ibid.

^228^ Guadamuz and Cabell (n 25).

^229^ ibid.

^230^ Meys (n 184).

^231^ ibid.

^232^ Rosati (n 13).

^233^ ibid.

^234^ ibid.

^235^ ibid.

^236^ ibid.

^237^ ibid.

^238^ ibid.

^239^ ibid.

^240^ Catherine Hammon and others, ‘What Risks Need to Be Considered by a Business Using Artificial Intelligence?’ (2023) 12 Compliance & Risk.

^241^ ibid.

^242^ ibid.

^243^ ibid.

^244^ ibid.

^245^ ibid.

^246^ Sean Fiil-Flynn, ‘Copyright and Generative AI: Insights from the Project on the Right to Research’ [2023] Joint PIJIP/TLS Research Paper Series 2 < https://digitalcommons.wcl.american.edu/research/112>.

^247^ Fiil-Flynn (n 246).

^248^ ibid 2–3.

^249^ ibid 3.

^250^ ibid 3–4.

^251^ ibid 4.
