# Supplementary material for: A literature review of “lawful” text and data mining
Source: Open Res Eur. 2024 Oct 30;4:153. Originally published 2024 Jul 24. [Version 2] doi: 10.12688/openreseurope.18013.2 (PMC11535487; doi:10.12688/openreseurope.18013.2)
Supplement: Peer review history

# Reviewer report: Reviewer response for version 2

**DOI:** 10.21956/openreseurope.20215.r45724

**Published:** 2024-11-04

**Authors:** Ana Nordberg

**Affiliations:** 1 Lund University, Lund, Sweden

I have no further comments.

Are all factual statements correct and adequately supported by citations?

Partly

Is the review written in accessible language?

Partly

Are the conclusions drawn appropriate in the context of the current research literature?

Yes

Is the topic of the review discussed comprehensively in the context of the current literature?

Yes

Reviewer Expertise:

Intellectual Property Law; Data protection; Technology regulation.

I confirm that I have read this submission and believe that I have an appropriate level of expertise to confirm that it is of an acceptable scientific standard.

# Reviewer report: Reviewer response for version 1

**DOI:** 10.21956/openreseurope.19468.r42476

**Published:** 2024-08-12

**Authors:** Pablo Rodrigo Trigo Kramcsak

**Affiliations:** 1 Vrije Universiteit Brussel, Brussels, Brussels, Belgium

I. Overview

 The paper offers a valuable literature review on Text and Data Mining (TDM), mapping the complex regulatory framework surrounding these processes and shedding light on what constitutes lawful TDM. However, several areas require improvement for the paper to reach its full potential. This report provides some comments and suggestions to strengthen the paper's structure, argumentation, and use of sources.

 II. Comments

 1) Introduction

  

- Structure improvement: The introduction could be better structured to present the study's subject matter clearly. The first two paragraphs are not sufficiently clear, particularly in introducing the concept of "big data." It would be beneficial to discuss the characteristic elements of big data and differentiate between structured, unstructured, and semi-structured data. This is crucial for readers to grasp the subject thoroughly. For reference, see Jonathan Grossman and Ami Pedahzur, [Ref:1]

 

- Clarification of concepts: The sentence ‘data “is the new oil and lifeblood of economic development” and just as oil needs to be burnt for it to be useful, raw data must be processed to realise its true potential’ is problematic and contested. This analogy oversimplifies the complexities of data processing in the digital economy. See, e.g., Clemens Mewald, ‘Why Data Is *Not* the New Oil and Data Marketplaces Have Failed Us’ (Medium, 13 July 2023) https://towardsdatascience.com/why-data-is-not-the-new-oil-and-data-marketplaces-have-failed-us-b42dd87a0ba0; David Moschella, ‘Data Isn’t the New Oil; That Might Be a Good Thing’ (2023) https://itif.org/publications/2023/05/30/data-isnt-the-new-oil-that-might-be-a-good-thing/

2) Regulatory Framework

 A broader perspective is needed: The paper should more comprehensively address the regulatory challenges of TDM, particularly from a data protection standpoint. This includes not only the technical challenges of big data processing but also the legal issues associated with scraping and mining of publicly available data. Including opinions from public agencies and supervisory bodies would offer a more holistic view of the regulatory landscape. These perspectives could be instrumental in identifying the main challenges to individual rights and public interests.

 3) Data Protection Issues

  

- Insufficient coverage of GDPR issues: The paper does not sufficiently address the complexities and challenges that data mining poses for data subjects. The paper relies on some sources of questionable quality. It would benefit from a more careful selection of references, particularly those recognised as authoritative in the field. I recommend citing more reliable sources, especially when describing the fundamental elements of GDPR, such as Christopher Kuner and others (eds), [ *Ref:2],A Commentary* (New York, 2020; online edn, Oxford Academic); Indra Spiecker genannt Döhmann and others (eds), *General Data Protection Regulation: Article-by-article Commentary* (München, 2023; Beck).

- Concept of "meaningful research": The paper should elaborate on the idea of "meaningful research" and how data protection regulations address data processing for scientific, research, or statistical purposes. This is a critical aspect that currently needs more depth.

 

- Data Protection by Design and Default: When discussing the implementation of the principle of data protection by design and default, it is important to reference guidelines issued by supervisory authorities. For instance, the EDPB Guidelines 4/2019 on Article 25 Data Protection by Design and by Default [Ref 5]; Commission Nationale d’Informatique et des Libertés (CNIL), GDPR guide for developers  (2022); Agencia Española de Protección de Datos (AEPD), A Guide to Privacy by Design (2019).

 

- Data mining, profiling and inferences: The paper should more comprehensively address the challenges and risks of data mining, especially concerning profiling and the processing of inferred data. The relevance of purpose limitation and data minimisation principles needs more emphasis. Key references include Bart Custers and others (eds), [ *Ref*:3,4]

 

- Missing legal aspects. Data scraping: The paper currently lacks a discussion on the legal aspects of data scraping at scale and text mining of publicly available data, which are increasingly relevant, especially for training AI models. It would be beneficial to incorporate an analysis of the legal challenges posed by these practices, e.g., Benjamin Bremert, *Legal Aspects of Text Mining Publicly Available Data*, Unabhängiges Landeszentrum für Datenschutz (ULD), 2017; Zuzanna Gulczynska, ‘Scraping personal data from internet pages - a comparative analysis of the Polish Bisnode decision and the US hiQ Labs v LinkedIn Corp judgment’, European Law Review, vol. 45, no. 6, pp. 857–869, 2020; Catherine Altobelli and others, ‘To Scrape or Not to Scrape? The Lawfulness of Social Media Crawling under the GDPR’, in Jean Herveg (ed), *Deep Driving into Data Protection: 1979-2019: Celebrating 40 Years of Privacy and Data Protection at the CRIDS* (Larcier, 2021); Autoriteit Persoonsgegevens, Richtlijnen scraping door private organisaties en particulieren (2024)

4) TDM and fundamental rights

 Effectively addressing bias and discrimination in data mining is critical to safeguarding fundamental rights. Failure to do so can result in inequitable outcomes that disproportionately harm marginalised groups, perpetuating existing social inequalities. See Bettina Berendt and Sören Preibusch, ‘Toward Accountable Discrimination-Aware Data Mining: The Importance of Keeping the Human in the Loop-and Under the Looking Glass’, Big Data. 2017 Jun;5(2):135-152; Solon Barocas and Andrew Selbst, ‘Big Data's Disparate Impact’, 104 California Law Review 671 (2016); Solon Barocas, ‘Data Mining and the Discourse on Discrimination’, Conference on Knowledge Discovery and Data Mining (2014).

 5) Several footnotes and cross-references need to be revised and improved for accuracy and clarity.

Are all factual statements correct and adequately supported by citations?

Partly

Is the review written in accessible language?

Partly

Are the conclusions drawn appropriate in the context of the current research literature?

Partly

Is the topic of the review discussed comprehensively in the context of the current literature?

Yes

Reviewer Expertise:

Personal data protection, data governance, AI regulation

I confirm that I have read this submission and believe that I have an appropriate level of expertise to confirm that it is of an acceptable scientific standard, however I have significant reservations, as outlined above.

## References

[1] : Political Science and Big Data: Structured Data, Unstructured Data, and How to Use Them. *Political Science Quarterly* .2020;135(2) : 10.1002/polq.13032 225-257 10.1002/polq.13032

[2] The EU General Data Protection Regulation (GDPR).2020; 10.1093/oso/9780198826491.001.0001 10.1093/oso/9780198826491.001.0001

[3] Discrimination and Privacy in the Information Society /authors.

[4] A Right to Reasonable Inferences: Re-Thinking Data Protection Law in the Age of Big Data and AI/authors.

# Response

**Published:** 2024-10-02

**Authors:** Giorgos Vrakas

**Affiliations:** Law, University of Cyprus, Nicosia, Nicosia, Cyprus

Thank you very much for your constructive and insightful comments. I have thoroughly revised the paper in light of your feedback, and I believe these changes have significantly strengthened the review. Below, I address each of your comments and explain the adjustments made.

1. **Introduction: Structure and Clarity**

I have restructured the introduction to improve clarity. The concept of "big data" is now introduced with a more thorough explanation of its characteristic elements, including a clearer distinction between structured and unstructured data. I have also reconsidered the analogy of data being “the new oil,” and, following your advice, I have removed  such statements.

1. **Regulatory Framework: Broader Perspective**

The section on the regulatory framework has been expanded to offer a broader and more comprehensive discussion of the legal challenges associated with TDM, particularly from a data protection standpoint. I have added references to opinions from public agencies and supervisory bodies to provide a more holistic view of the regulatory landscape. **Data Protection Issues: More Depth and Careful Source Selection** The data protection section has been substantially reworked to include more authoritative and recognised sources, as you recommended. Additionally, I have expanded on the concept of "meaningful research," elaborating on how data protection regulations address the processing of data for scientific, research, or statistical purposes. I have also incorporated references to the EDPB Guidelines 4/2019 on Article 25 (Data Protection by Design and Default), as you suggested. **Data Mining, Profiling, and Inferences** I have significantly expanded the discussion on the challenges and risks of data mining, particularly regarding profiling and the processing of inferred data. **Legal Aspects of Data Scraping** I have added a paragraph discussing the legal aspects of data scraping citing the sources that you had recommended.

1. **Fundamental Rights: Bias and Discrimination**
  - I have reworked the section on fundamental rights to more thoroughly address the critical issues of bias and discrimination in data mining.
2. **Footnotes and Cross-References**
  - I have revised the footnotes and cross-references to improve their accuracy and clarity. This includes correcting any inconsistencies and ensuring that all references are properly cited and linked to the relevant sections of the paper.

Once again, thank you for your detailed feedback. I am confident that the revisions made in response to your comments have strengthened the paper both in terms of structure and substantive content. 

# Reviewer report: Reviewer response for version 1

**DOI:** 10.21956/openreseurope.19468.r42474

**Published:** 2024-08-07

**Authors:** Ana Nordberg

**Affiliations:** 1 Lund University, Lund, Sweden

The paper provides a highly valuable overview of the literature on the topic of  TDM.  Exploring the literature in a meaningful manner and highlighting scholarly work conducted  on this topic.

 The following comments and suggestions are submitted for the author’s consideration:

 Methodology  

 The sources included in the review are mostly relevant and the analysis is sound however some aspects of the methodology section would benefit from further development:

  

1. The sources selected consist mostly of well-known authors and reputable publication venues. However, the characteristics of journals or other venues of publications either were not considered or are not described. Given the proliferation of low-quality, biased or otherwise unreliable publications, it should be addressed whether or not the study included a qualitative approach to  the selection of sources, and if relevant how.
2. Table 1 categorizes the literature by topics (most of them of a legal nature), however, some of the sources appear to present other disciplinary perspectives.  It is unclear whether the field of research of the literature analyzed was considered and how such influenced the qualitative analysis of the materials.
3. A large part of the literature analyzed is legal literature where the jurisdictional aspect is relevant, the methodology section does not clearly state the jurisdictional focus of the study and whether comparative law or other methodologies were used in the qualitative analysis. Instead, the section ‘jurisdictional variance’ provides some information in this regard which could be more detailed.
4. Arguably, there is a strong possibility that a considerable body of legal literature has been published in the official languages of each jurisdiction, in particular in those countries that have enacted national legislation on TDM that predates Directive (EU) 2019/790 CDSM. However, the literature reviewed only included publications in the English language. If language was a selection factor it should be described and treated as an important methodological limitation or has a subject scope delimitation.

Readability

1. The text is well written with occasional less clear sentences. Some sentences and paragraphs are excessively long and complex.
2. The use of the plural ‘they’ to avoid gender pronouns can be confusing. Gender neutrality is important and can be better achieved with the person's name or neutral expressions, such as ‘the author’  or equivalent.
3. A few footnotes require revision and the entries in the appendix are inconsistently cited, with many being incomplete.

These points can easily be improved with the usual final text review, which is recommended. 

 Substantive content

 1.Section ‘Regulatory frameworks which govern TDM'

  Would benefit from a clear structure and a definition of what is understood by “lawful TDM”. The term lawful entails a presumption of compliance with a set of legal rules, thus it should be accompanied by clear specification of the said set of rules (including eventual conditions and exceptions).

 2. Section ‘TDM and Intellectual Property Rights’

 The section does not address all IPRs, perhaps it would benefit from being more accurately named or at least the type of IPRs considered could be enunciated in the section's introductory sentences.

  Some statements in the text are excessively vague or not sufficiently explored/supported. For example, the statement below introduces several points of comparative law that are left ‘hanging in the air’,  and are not explored further in the section.  I would suggest either erasing ( ‘kill the darling’) or further elaborating.

 ‘Jackson Bertón, who examined the applicable copyright frameworks of the five largest economies of South America in a pre-CDSM era, claimed that even then, the EU was falling behind other more competitive jurisdictions, but ultimately found that South American frameworks were not prepared for digital research techniques.’

 3. Section ‘ TDM and Data Protection’

 Here the reader faces difficulties with the jurisdictional aspect. Although the information is implied, it should be made more clear what is the  jurisdictional origin and coverage of the legal instruments mentioned (e.g. GDPR). It is not always clear what jurisdiction was considered in the papers analysed.

 Some of the papers analysed in this section were written in the early 2000’s. The temporal aspect could be better scrutinized  as to whether their findings remain unchanged by subsequent technological and legal developments.

 4. Section ‘TDM and  Fundamental Rights’

  What is understood by fundamental rights and what legal framework(s) are considered is not defined. The expressions ‘fundamental rights’, ‘human rights’ and ‘fundamental human rights’ are sometimes used interchangeably making those sentences confusing.

 5.Section ‘Generalist approach’

 The qualification of the papers analysed under this section as ‘generalist’ is not very convincing. Perhaps the author could better justify their classification, or otherwise include this literature in previous sections.

Are all factual statements correct and adequately supported by citations?

Partly

Is the review written in accessible language?

Partly

Are the conclusions drawn appropriate in the context of the current research literature?

Yes

Is the topic of the review discussed comprehensively in the context of the current literature?

Yes

Reviewer Expertise:

Intellectual Property Law; Data protection; Technology regulation.

I confirm that I have read this submission and believe that I have an appropriate level of expertise to confirm that it is of an acceptable scientific standard, however I have significant reservations, as outlined above.

# Response

**Published:** 2024-10-02

**Authors:** Giorgos Vrakas

**Affiliations:** Law, University of Cyprus, Nicosia, Nicosia, Cyprus

Thank you for your constructive feedback, which has been invaluable in helping me to strengthen this literature review. I have carefully addressed each of your comments and made the necessary revisions to the paper. Below, I provide a detailed response to your suggestions.

1. **Methodology: Selection Criteria and Language Limitation**

I have clarified the selection criteria for the literature included in this review. The introduction now explicitly discusses the section criteria used. I have also acknowledged that the study focused on publications in English due to the scope of the review, and I have identified this as a methodological limitation. Additionally, I have noted the possibility that significant legal literature may exist in other languages, particularly in countries with pre-existing TDM legislation, and this is now treated as a subject scope limitation.

1. **Use of ‘They’ and Readability**

Following your suggestion, I have replaced the use of the plural ‘they’ with specific references to ‘the author’ or the author's name to avoid confusion. I have also restructured several sections, shortening complex and overly long sentences to improve readability and clarity. A review of the footnotes has been conducted to correct inconsistencies, and the appendix has been updated to ensure all entries are complete and correctly cited.

1. **Definition of “Lawful TDM” in Regulatory Frameworks Section**

I have revised the section on regulatory frameworks to include a clearer definition of “lawful TDM.” The term is now defined as “TDM that is considered compliant with applicable regulatory frameworks”. This should make the concept more transparent for the reader.

1. **TDM and Intellectual Property Rights Section**

Based on your feedback, I have renamed the section ‘TDM and Intellectual Property Rights’ to ‘TDM and Copyright and Related Rights’ to better reflect its content. Furthermore, I have removed the portions that you suggested did not add sufficient value to the discussion, such as the statement regarding Jackson Bertón’s analysis of South American copyright frameworks. This has helped to eliminate vague or unsupported statements, ensuring the section remains focused and well-supported.

1. **Fundamental Rights Section**

I have revised the section on TDM and Fundamental Rights to clarify what is meant by fundamental rights. The introductory paragraphs now define the legal frameworks considered, and I have ensured that the terms ‘fundamental rights’, ‘human rights’, and ‘fundamental human rights’ are used consistently to avoid confusion. This should provide a more structured and coherent discussion.

1. **Generalist Approach Section**

I have revisited the ‘Generalist Approach’ section, and I now provide a clearer justification for the classification of the papers analysed under this category. I explain why they are considered ‘generalist’ in their approach to TDM, distinguishing this category from other sections of the review. Once again, thank you for your insightful suggestions. These revisions have contributed significantly to improving the clarity, structure, and overall quality of the paper. 

# Reviewer report: Reviewer response for version 1

**DOI:** 10.21956/openreseurope.19468.r42477

**Published:** 2024-07-30

**Authors:** Thomas Margoni

**Affiliations:** 1 KU Leuven, Leuven, Belgium

This is a very relevant piece of research reviewing the literature in the field of TDM. As such it is important and, within the limits of the initial stage of the research project, original and well developed. A few observations can be found below that may be taken into considerations by the author in the next stages.

 "Lawful tdm": not clear what it is, lacks definition.

 Literature review: not clear in which disciplinary domains it was conducted. Are you assessing mainly legal sources, or also technical literature (computer science; digital humanities, etc)? There seems to be a need to define better the boundaries of the review and which concepts are assumed.

 “Some scholars make a distinction between text mining and data mining. ^18^ Whilst this distinction may be vital on the technical side of TDM, altering the analysis techniques used, it arguably does not necessarily play a pivotal role in the regulatory frameworks which may apply. Data mining is sometimes seen as a subset of text mining since text mining is the computational process of analysing unstructured messy data, whilst data mining is the computational process of analysing structured data. ^19^ “

 General observation: It is not always clear where you are reviewing current literature and where you are taking a position in the debate. This should be made clearer. As a lit. rev. I would assume the former, but I have doubts here and there.

 E.g.,: One could of course argue that text is more copyright focused, while data is less copyright and, if applicable, more sgdr/neighbouring rights with different regulatory impact. Is this something that you are addressing?

 Lit rev. may need to be temporally structured: e.g., sources of 2022-2024 are discussed together with sources of 2014 (e.g., around fn 20 – 25 but also in other parts), but the underlying technology and regulatory framework have evolved considerably. Are there category commensurate?

 “Bearing in mind the definition of TDM presented above” why not using the CDSM definition?

 “One of these solutions, Manteghi claims could be found under Article 35 of the proposed Data Act which “ *provides a safe harbour for users needing to access, use or share only databases made of data generated by the use of a product or related service (IoT data)”. ^83^* “

 Unclear. Perhaps clarify that Art. 43 (ex 35) Data Act, only refers to SGDR not copyright.

 “On the other hand, Kretschmer *et al.*, who examined machine learning, natural language processing and computer vision for computer moderation as case studies where TDM is vital, found that legal uncertainties like those created by fair use exceptions, can lead to practices exploiting these uncertainties for commercial gain, particularly by large tech corporations.“

 Same as above.

 General observation: It seems that certain statements are somehow decontextualised from the original source and used in a way that changes the meaning. In the specific example above, it seems that Kretschmer is against fair use for tdm. If I recall it correctly, they are in favour, however, they suggest that legal uncertainty (which can be found in various legal mechanisms including fair use) has, among its consequences, that of favouring larger players who have the financial resources to litigate/resist in court.

 It may be necessary to revise that all the excerpts are correctly contextualised in order to ensure that the cited literature is assessed properly.

 The part on fundamental right seems a bit rushed in comparison to copyright and DP and to conflate EU/US?

 The “generalist approach” category and section is not clear. How is it different from the sections analysed above, particularly copyright? It also seems to rediscuss some of the same sources already analysed.

 Conclusions: again it seems you are expressing an opinion about TDM, but can you do that on the basis of a lit. rev.? Perhaps, at this stage you should only focus on what emerges from lit. rev. (which you do in part at the end of the section).

Are all factual statements correct and adequately supported by citations?

Partly

Is the review written in accessible language?

Partly

Are the conclusions drawn appropriate in the context of the current research literature?

Partly

Is the topic of the review discussed comprehensively in the context of the current literature?

Yes

Reviewer Expertise:

IP, copyright, TDM.

I confirm that I have read this submission and believe that I have an appropriate level of expertise to confirm that it is of an acceptable scientific standard, however I have significant reservations, as outlined above.

# Response

**Published:** 2024-10-02

**Authors:** Giorgos Vrakas

**Affiliations:** Law, University of Cyprus, Nicosia, Nicosia, Cyprus

Thank you for your valuable feedback. I have carefully considered each of your comments and made revisions accordingly to improve the clarity and strength of the paper. Below, I provide a point-by-point response to your suggestions.

1. **"Lawful TDM" is unclear, lacks definition:**

I have now briefly defined “lawful TDM” as “TDM which would be considered compliant with the applicable regulatory frameworks” in the relevant section, ensuring that this term is clearer for the reader.

1. **Literature review: disciplinary domains unclear:**

I have clarified in the introduction that no disciplinary boundaries were imposed in the selection criteria for the literature reviewed, to reflect the interdisciplinary nature of TDM. This literature spans legal, technical, and policy domains. This revision aims to better communicate the broad scope of the review.

1. **Clarification of position vs. review:**

I have made clearer distinctions between my own interpretations and the positions found in the literature. Where I offer commentary, it is now more clearly framed as a response or evaluation of the literature, rather than presenting it as a standalone position. This is particularly important for a literature review, and I appreciate your observation in this regard.

1. **Temporal structuring of the literature:**

Although I have chosen not to temporally structure the review, I have made it clearer when older sources are discussed alongside newer ones. I have provided explicit justification for the inclusion of these older sources, explaining why their contributions remain relevant despite technological and regulatory developments.

1. **Use of CDSM definition:**

I have revised the relevant section to incorporate the CDSM definition of TDM, as you suggested. This revision ensures greater coherence with existing EU legal frameworks.

1. **Clarification regarding Article 43 (formerly Article 35) of the Data Act:**

I have clarified that Article 43 (formerly 35) of the Data Act refers specifically to SGDR, not copyright, as you rightly pointed out.

1. **Contextualisation of Kretschmer et al.'s position on fair use:**

I have revisited the discussion of Kretschmer et al.'s work to ensure that it is accurately contextualised. The revision better reflects their nuanced position on fair use, highlighting their concern about legal uncertainty benefiting larger players, while also ensuring that their general support for fair use is not misrepresented.

1. **Fundamental rights section and conflation of US/EU perspectives:**

I have revised the fundamental rights section to avoid conflating EU and US perspectives. The revision now presents these frameworks more distinctly.

1. **Generalist approach and redundancy in the review:**

I have restructured the “generalist approach” section to avoid redundancy, ensuring that it builds upon prior sections rather than repeating the same sources or arguments. I have clarified how it differs from the copyright-focused discussions, making the overall structure of the paper more coherent.

1. **Conclusions:**

I have revised the conclusion to ensure that it focuses strictly on summarising the key findings from the literature review. I have removed any personal opinions or evaluative commentary that could be construed as going beyond the scope of a literature review.   Thank you once again for your insightful comments, which have contributed significantly to refining the paper.
